# Red Light-Driven, Oxygen-Tolerant RAFT Polymerization Enabled by Methylene Blue

**DOI:** 10.1021/jacs.5c10541

**Published:** 2025-08-21

**Authors:** Lucca Trachsel, Ivan O. Levkovsky, Xiaolei Hu, Hironobu Murata, Krzysztof Matyjaszewski

**Affiliations:** Department of Chemistry, 6612 Carnegie Mellon University, Pittsburgh, Pennsylvania 15213, United States

## Abstract

Photoinduced reversible-deactivation radical polymerization (photoRDRP) techniques enable the synthesis of well-defined polymers under mild conditions. However, oxygen inhibition remains a key challenge, requiring either complex deoxygenation methods or *in situ* oxygen-scavenging strategies. Here, we report a red light-driven, fully oxygen-tolerant reversible addition–fragmentation chain transfer (RAFT) polymerization mediated by methylene blue (MB^+^) as a photosensitizer and triethanolamine (TEOA) as an electron donor. This operationally simple method enables polymerization in fully open-to-air vials without stirring, even under direct sunlight, underscoring the robustness and accessibility of the system. Using MB^+^ in the presence of a sacrificial electron donor, the polymerization proceeds to high monomer conversions (>90%), with excellent temporal control, predictable molecular weights, and low dispersities (*Đ* < 1.3) across a wide range of conditions. The system is compatible with a broad scope of hydrophilic (meth)­acrylamide and (meth)­acrylate monomers, including those bearing charged and zwitterionic side groups. Notably, this methodology enables access to ultrahigh molecular weight (UHMW, >1,000,000) polymers under ambient conditions, an outcome rarely achieved in oxygen-tolerant RDRP. This metal-free, red light-driven photoRAFT platform offers a scalable, efficient, and biocompatible strategy for controlled polymer synthesis, with potential applications in bioconjugation, functional coatings, and high-throughput screening.

## Graphical abstract

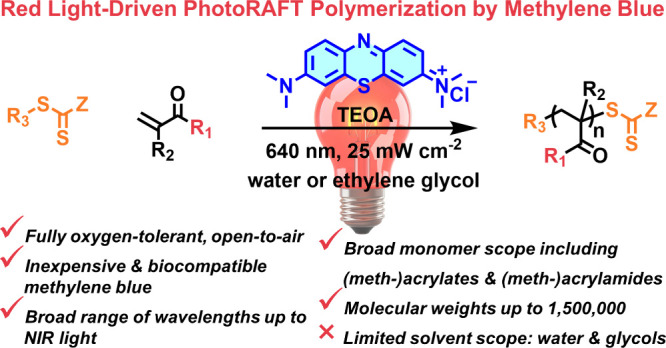

## Introduction

The use of light to mediate chemical transformations has become a powerful tool in synthetic chemistry due to its tunability, noninvasive nature, and ability to provide spatiotemporal control.^1,2^ Reversible-deactivation radical polymerization (RDRP) techniques^3,4^ enable the synthesis of well-defined polymers and complex hybrid materials with precise control over molecular weight and architecture.^5–7^ Among the most widely studied photoinduced RDRP (photoRDRP) methods^8,9^ are photoinduced atom transfer radical polymerization (photoATRP),^10,11^ photocontrolled reversible addition–fragmentation chain transfer (RAFT) polymerization,^12–14^ and photoiniferter polymerization.^15^ These strategies have enabled ambient-temperature polymerization of a broad range of vinylic monomers, offering enhanced spatiotemporal control and opportunities for high-throughput and automation,^16–18^ 3D printing,^7^ and bioconjugation.^19–22^ However, challenges remain, particularly with respect to oxygen sensitivity, limited light penetration in high-throughput or biological contexts, and compatibility with aqueous or complex media.^23–25^ As such, the development of oxygen-tolerant, metal-free, and operationally simple photoRDRP platforms that function under red or near-infrared (NIR) light remains a critical goal in advancing precision polymer synthesis.

RAFT polymerization is attractive due to its broad monomer compatibility, functional group tolerance, and operational simplicity.^26,27^ However, oxygen remains a major obstacle in radical polymerizations, including RAFT, as it efficiently quenches radical intermediates,^28^ necessitating stringent deoxygenation methods, such as inert-gas purging or freeze–pump–thaw cycles, which can be time-consuming, technically challenging, and difficult to scale or automate. To address this, several oxygen-tolerant RAFT systems have emerged,^23^ including enzyme-mediated RAFT,^29–31^ Fenton RAFT,^32–34^ organoborane-initiated systems,^35,36^ and photomediated methods.^37,38^ In particular, photoinduced electron/energy transfer (PET)-RAFT polymerization has been widely explored using photoredox catalysts capable of simultaneously generating initiating radicals and reducing molecular triplet oxygen to less reactive species, allowing polymerization without extensive deoxygenation.^39,40^ However, many of these methods require expensive or metal-based photocatalysts, or coadditives like DMSO, which may limit biocompatibility, scalability, or use in sensitive environments.^41^

Oxygen-tolerant photoinitated RAFT (photoRAFT) systems have also been developed to mitigate these limitations. A green-light-driven photoRAFT polymerization using Eosin Y and ascorbic acid was reported, enabling aqueous polymerizations in open-air conditions (Figure 1A).^42,43^ More recently, a sodium pyruvate-assisted photoRAFT approach driven by UV to green light was introduced, offering enhanced oxygen tolerance and biocompatibility but limited in accessible molecular weights and scope (Figure 1B).^44^ Building on these strategies, here we report a red-light-driven RAFT polymerization mediated by methylene blue (MB^+^) and triethanolamine (TEOA), which proceeds under fully open-air conditions—even in ambient sunlight—and enables access to ultrahigh molecular weight (UHMW, *M*_n_ > 1,000,000) polymers (Figure 1C).

**Figure 1:**
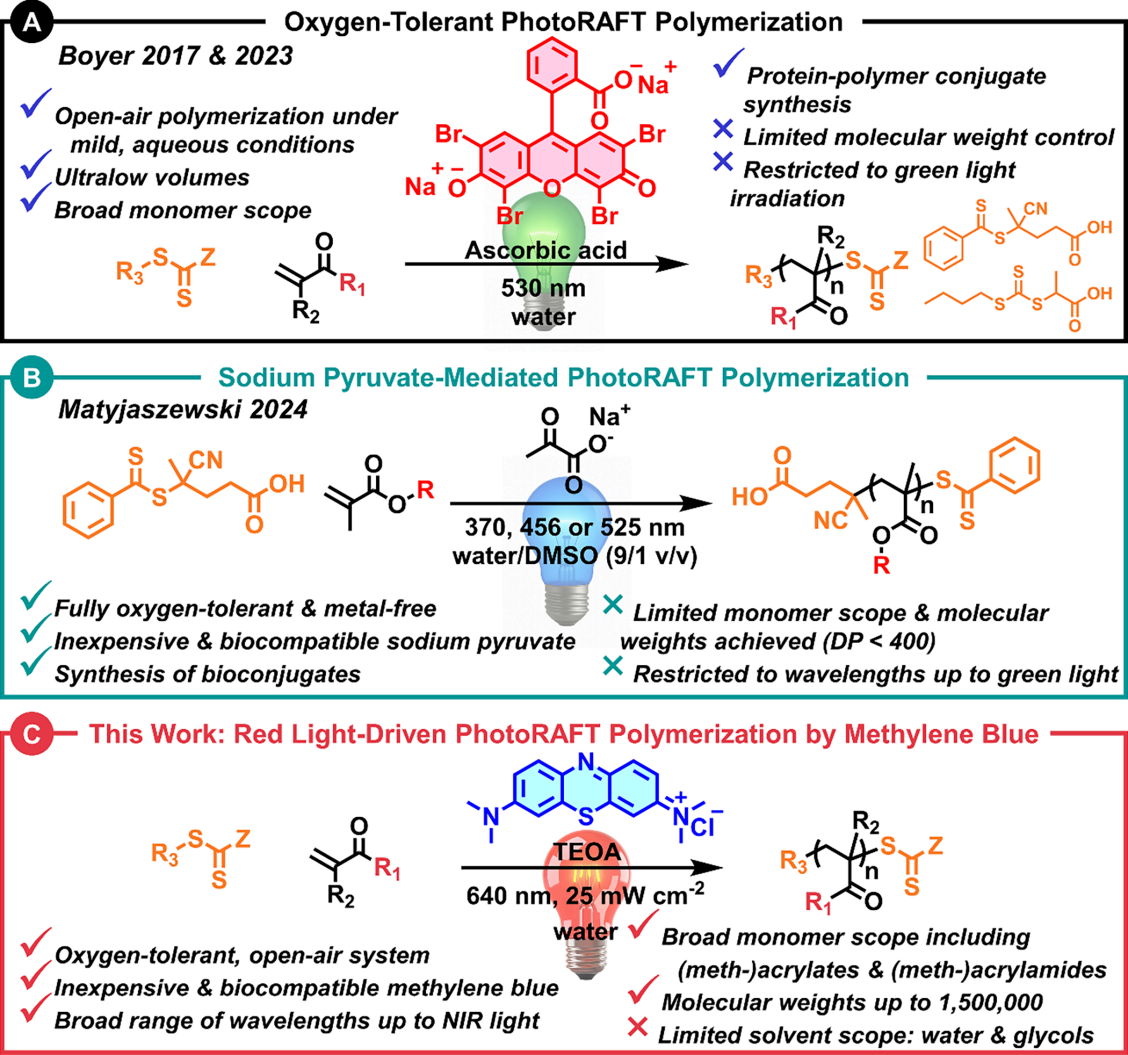
Overview of selected oxygen-tolerant photoinitated RAFT (photoRAFT) strategies. (A) Boyer et al. developed a green-light-driven photoRAFT system using Eosin Y and ascorbic acid, enabling polymerization in open-air aqueous environments with broad monomer scope, but limited to low wavelengths and ultralow volumes.^42,43^ (B) Matyjaszewski et al. reported a sodium pyruvate-mediated photoRAFT approach driven by UV to green light, offering full oxygen tolerance and bioconjugation compatibility, though with narrow monomer scope and restricted molecular weights.^44^ (C) This work: We present a red-light-driven, methylene blue (MB^+^)/TEOA-mediated RAFT polymerization platform that is fully oxygen-tolerant, operational under ambient and even sunlight conditions, compatible with a broad range of monomers, and capable of producing ultrahigh molecular weight polymers (*M*_n_ > 1,000,000).

Dye-sensitized photopolymerization offers a metal-free alternative for radical generation.^45,46^ MB^+^, a cationic, inexpensive, and biocompatible organic dye, has been explored as a photocatalyst in organic synthesis,^47,48^ and has long served as a photosensitizer in radical polymerizations when paired with electron donors such as TEOA.^49,50^ Recently, MB^+^ was utilized in dual-catalyzed photoATRP, where reversible redox cycling enabled efficient polymerizations under red and NIR irradiation (640–730 nm), enabling open-to-air polymerizations in both homogeneous and heterogeneous media.^51–53^ MB^+^-mediated photo-Fenton-like RAFT polymerizations have been reported, but these strategies rely on hydrogen peroxide addition, which complicates reaction conditions, can lead to oxidative degradation of sensitive substrates, and limits monomer scope.^54,55^

Inspired by these developments, we envisioned a distinct MB^+^/TEOA-mediated RAFT polymerization system that eliminates the need for hydrogen peroxide, metal catalysts, or other additives. In contrast to previous MB^+^-mediated photo-Fenton systems, our approach simplifies reaction conditions and improves compatibility with sensitive functional groups under mild, operationally practical conditions. Furthermore, unlike dual-catalyzed MB^+^-mediated photoATRP, this strategy avoids metal-based catalytic cycles entirely, making it particularly attractive for bioconjugation and biomedical applications.

Herein, we report a red-light- and sunlight-driven RAFT polymerization system mediated by MB^+^ and TEOA that operates entirely under ambient conditions without deoxygenation, fully open to air. We demonstrate broad monomer compatibility, scalability, and access to UHMW polymers with moderate dispersity. This metal-free, oxygen-tolerant platform provides a robust and accessible method for controlled polymer synthesis, offering practical utility in high-throughput screening, functional coatings, and biomaterials design.

## Results and Discussion

### Initial Optimization of PhotoRAFT Polymerization

We began our investigation into red-light-driven RAFT polymerization of *N*,*N*-dimethylacrylamide (DMA) mediated by MB^+^ and TEOA, inspired by previous reports on conventional free radical polymerization of acrylamide in aqueous solution photosensitized by a MB^+^-TEOA system.^49^ Initial polymerizations were conducted in capped glass vials without deoxygenation, using 2-(dodecylthiocarbonothioylthio)-2-methylpropanoic acid (DDMAT) as the chain transfer agent (CTA) at fixed [DMA]/[DDMAT] = 200/1 and [DMA] = 3.0 M in water/DMSO (9:1 v/v), under red-light irradiation (λ_max_ = 640 nm, 25 mW cm^–2^). At [MB^+^] = 150 μM and [TEOA] = 20 mM, high monomer conversion (90%) was achieved within 4 h and the resulting polymer exhibited low dispersity (*Đ* = 1.14) and good agreement between the theoretical number-average molecular mass determined by ^1^H NMR spectroscopy (*M*_n,theory_ = 17,800 g mol^–1^) and the apparent number-average molecular mass determined by SEC relative to poly­(methyl methacrylate) (PMMA) standards (*M*_n,app_ = 22,400 g mol^–1^) (entry 5, Table 1, Figure S1A). The discrepancy between theoretical and measured values can be explained by the difference in hydrodynamic volume between PDMA and PMMA standards in DMF. Using control experiments omitting MB^+^, TEOA, or light resulted in negligible monomer conversion (entries 2–4, Table 1). Additionally, performing the reaction without DDMAT led to an uncontrolled conventional free radical polymerization, yielding polymers with high molar mass and dispersity (*M*_n,app_ = 352 kg mol^–1^, *Đ* = 2.35) (entry 1, Table 1, and Figure S1A). These results confirmed the necessity of MB^+^, TEOA, red light, and CTA for controlled RAFT polymerization. Polymerizations conducted in uncapped vials fully open-to-air produced comparable outcomes, demonstrating the intrinsic oxygen tolerance of the MB^+^/TEOA photoRAFT system (entry 6, Table 1). Although initial experiments included DMSO (10 vol %) because of stock solutions made in DMSO, polymerizations in pure water (without DMSO) also yielded well-controlled polymers (*M*_n,app_ = 19,100 g mol^–1^, *Đ* = 1.17) (entry 7, Table 1). This demonstrates that RAFT polymerization with the MB^+^/TEOA photosensitizer system under red light does not require DMSO as an oxygen scavenger, in contrast to PET-RAFT systems where DMSO is typically essential for quenching singlet oxygen.^56,57^

**Table 1:** Optimization of Polymerization Conditions^a^

Entry	DDMAT (equiv)	[MB^+^] (μM)	[TEOA] (mM)	Conv. (%)^e^	*M*_n,theory_ (kg mol^–1^)^f^	*M*_n,app_ (kg mol^–1^)^g^	*Đ* ^g^
1	0	150	20	84	-	352	2.35
2	1	150	0	0	-	-	-
3	1	0	20	0	-	-	-
4^b^	1	150	20	0	-	-	-
5	1	150	20	90	17.8	22.4	1.14
6^c^	1	150	20	87	17.2	21.8	1.14
7^d^	1	150	20	84	16.7	19.1	1.17
8	1	150	0.1	9.0	1.87	n.d.	n.d.
9	1	150	1.0	28	5.50	7.71	1.23
10	1	150	10	82	16.3	21.2	1.15
11	1	150	20	95	18.9	23.6	1.13
12	1	150	50	>99	19.8	24.3	1.12
13	1	10	20	25	4.88	7.50	1.26
14	1	25	20	70	13.8	19.0	1.13
15	1	50	20	91	18.1	25.5	1.11
16	1	100	20	91	18.1	26.0	1.11
17	1	300	20	>99	19.8	28.1	1.16
18	1	450	20	>99	19.8	27.1	1.14

a Reaction conditions: [DMA]/[DDMAT]/[MB^+^]/[TEOA] = 200/1/x/x, [DMA] = 3.0 M, in water/DMSO (9/1 v/v), irradiated for 10 h under red LED (640 nm, 25 mW cm^–2^).

b Polymerization was performed in the dark.

c Polymerization was performed in an open-to-air vial.

d Polymerization was performed in water only (no DMSO).

e Monomer conversion determined by ^1^H NMR spectroscopy.

f Theoretical number-average molecular masses (*M*_n,theory)_ were determined from the monomer conversion from ^1^H NMR.

g Apparent number-average molecular masses (*M*_n,app_) and dispersity (Đ) were determined by SEC using DMF + 50 mM LiBr relative to poly­(methyl methacrylate) (PMMA) standards.

These results collectively suggest that the MB^+^/TEOA system is intrinsically oxygen tolerant and capable of maintaining polymerization control without additional additives.

We then investigated the effect of electron donor and photosensitizer concentrations by screening a range of [TEOA] = 0.1–100 mM and [MB^+^] = 10–450 μM (Table 1, Figure S1). Lowering [MB^+^] substantially reduced polymerization efficiency, with monomer conversions dropping to 25% at 10 μM and 70% at 25 μM MB^+^, both at 20 mM TEOA (entries 13 and 14, Table 1). Similarly, decreasing [TEOA] from 20 mM to 0.1 mM led to a progressive drop in conversion from 95% to 9.0% (entries 8–11, Table 1). These results confirm that both MB^+^ and TEOA must be present in sufficient concentrations to sustain effective radical generation and propagation.

Given the successful polymerization observed in a water/DMSO mixture, we next explored the solvent scope of the MB^+^/TEOA-mediated photoRAFT polymerization. In contrast to a previously reported MB^+^/hydrogen peroxide-based system that enabled polymerization in DMSO,^55^ our MB^+^/TEOA system showed no polymerization in pure DMSO, even after 10 h of red-light irradiation (entry 4, Table S1). This finding was particularly unexpected, as many recent photoRDRP methodologies,^58–61^ including PET-RAFT,^62,63^ perform optimally in DMSO due to its high polarity, favorable solvent properties, and its ability to quench singlet oxygen.^57^

To further assess solvent compatibility, we screened a variety of polar protic and aprotic solvents. Despite good MB^+^ solubility, polar protic solvents such as methanol and ethanol produced no monomer conversion (entries 7–8, Table S1). Encouraged by previously reported accelerated conventional free radical polymerization of acrylamide using MB^+^/TEOA in ethylene glycol compared to water,^64^ we also tested ethylene glycol in our system. Critically, polymerizations of DMA in ethylene glycol achieved monomer conversions and polymer characteristics comparable to those obtained in water or water/DMSO (entry 9, Table S1), but without the previously observed kinetic acceleration. Additionally, commonly used polar aprotic solvents in RAFT polymerization, including 1,4-dioxane, THF, DMF, and acetonitrile, resulted in no detectable monomer conversion, despite sufficient MB^+^ solubility (entries 10–14, Table S1).

To probe whether ground-state optical properties might explain these trends, we measured UV–Vis spectra of MB^+^ in various solvents (Figures S13–S14). Slight shifts in λ_max_ and variations in molar absorptivity were observed; however, neither parameter correlated with polymerization success. We also examined the recovery of MB^+^ absorbance after photoreduction in different solvents (Figures S15–S16). In water and ethylene glycol, partial recovery of MB^+^ absorbance was observed, whereas no recovery occurred in DMSO, DMF, or acetone. While this absence of recovery broadly coincided with a lack of polymerization activity, ethanol represented an exception—showing partial recovery but no polymerization—indicating that additional factors beyond MB^+^ ground-state spectra or simple redox reversibility must contribute to solvent-dependent reactivity. These may include differences in aggregation state, excited-state dynamics, or solvent–radical interactions.

Overall, these findings highlight that MB^+^/TEOA photoRAFT polymerization is highly sensitive to the solvent environment, proceeding efficiently only in water, water/DMSO mixtures, and ethylene glycol. This behavior cannot be explained by solubility alone—for example, although MB^+^ is more soluble in methanol than in water,^65^ polymerization is unsuccessful in methanol (Table S1, entry 8). One possible factor is the extent to which MB^+^ exists in its photoreducible monomeric form, as MB^+^ is known to form nonphotoreducible dimers in certain solvents,^66,67^ and ethylene glycol has been proposed to disrupt such aggregation.^64^

We also evaluated other trithiocarbonate (TTC)-based CTAs suitable for DMA polymerization: 2,2’-(carbono­thio­yl­disulfanediyl)­bis­(2-methylpropanoic acid) (CMP) and 3-((((1-carboxyethyl)­thio)­carbonothioyl)­thio)­propanoic acid (CETPA). CMP, which shares the same R-group as DDMAT, yielded high monomer conversion (>95%), good agreement between theoretical and measured molar masses, and low dispersity (*M*_n,theory_ = 19,400 g mol^–1^, *M*_n,app_ = 21,800 g mol^–1^, *Đ* = 1.10)—comparable to results obtained with DDMAT (entry 18, Table S1). In contrast, polymerization with CETPA (bearing a monomethyl instead of dimethyl R-group) led to significantly lower conversion and poor control (entry 17, Table S1), despite CETPA’s known suitability for DMA polymerization under other RAFT conditions.^68^ The MB^+^/TEOA system also displayed strong pH sensitivity. No polymerization occurred under acidic conditions (pH = 3), even after prolonged irradiation (10 h), while neutral to slightly basic conditions enabled efficient polymerization (entries 15–18, Table S1). This pH dependence likely reflects suppressed formation of the α-aminoalkyl radical at low pH due to inhibited α-C–H deprotonation of the amine radical cation intermediate (R_3_N^+•^).^69,70^

We further evaluated the influence of various tertiary amines as electron donors in the MB^+^/amine photoRAFT polymerization of DMA (Table S2, Figure S2C). Among the amines tested, TEOA provided the highest monomer conversion (95%), along with good agreement between theoretical and apparent molecular weights, and a low dispersity (*Đ* = 1.16). Other tertiary amines, including *N*-methyl-*N*,*N*-diethanolamine (MDEA), *N*,*N*-diisopropylethylamine (DIPEA), and tris­(2-dimethylaminoethyl)­amine (Me_6_TREN), showed comparable or slightly lower conversions (85–94%) and good polymerization control (*Đ* ≤ 1.21). In contrast, triethylamine (TEA) and tris­(2-pyridylmethyl)­amine (TPMA) resulted in notably lower conversions (31% and 41%, respectively) and broader molecular weight distributions. Notably, although an earlier report indicated that DIPEA was ineffective in generating α-aminoalkyl radicals,^71^ our results clearly demonstrate its competence as both a reducing agent for excited MB^+^ and as a source of radicals, achieving polymerization outcomes comparable to TEOA under our reaction conditions (Table S2).

### Kinetic Study

Using optimized conditions ([DMA]/[DDMAT]/[TEOA]/[MB^+^] = 200/1.00/1.33/0.01), we evaluated the polymerization kinetics (Figure 2A). SEC analysis revealed clean, monomodal shifts of the traces toward shorter elution times, indicating uniform chain growth throughout the polymerization (Figure 2B). A linear pseudo- first-order kinetic plot revealed constant radical concentration throughout the reaction, with an apparent propagation rate constant (*k*_p,app_ = 1.54 × 10^–4^ s^–1^) (Figure 2C). Additionally, *M*_n,app_ increased linearly with monomer conversion, although it deviated substantially from *M*_n,theory_, which is attributed to differences in the hydrodynamic volume of PDMA compared to the PMMA standards used for SEC calibration (Figure 2D). To assess the role of oxygen, we repeated the same polymerization under deoxygenated conditions, maintaining all other parameters. An approximately 30% slower polymerization (*k*_p,app_ = 1.07 × 10^–4^ s^–1^) was observed (Figure 2C), supporting the hypothesis that oxygen can regenerate photoactive MB^+^ from reduced leucomethylene blue (LMB), thereby sustaining the catalytic cycle and enhancing propagation under aerobic conditions.

**Figure 2:**
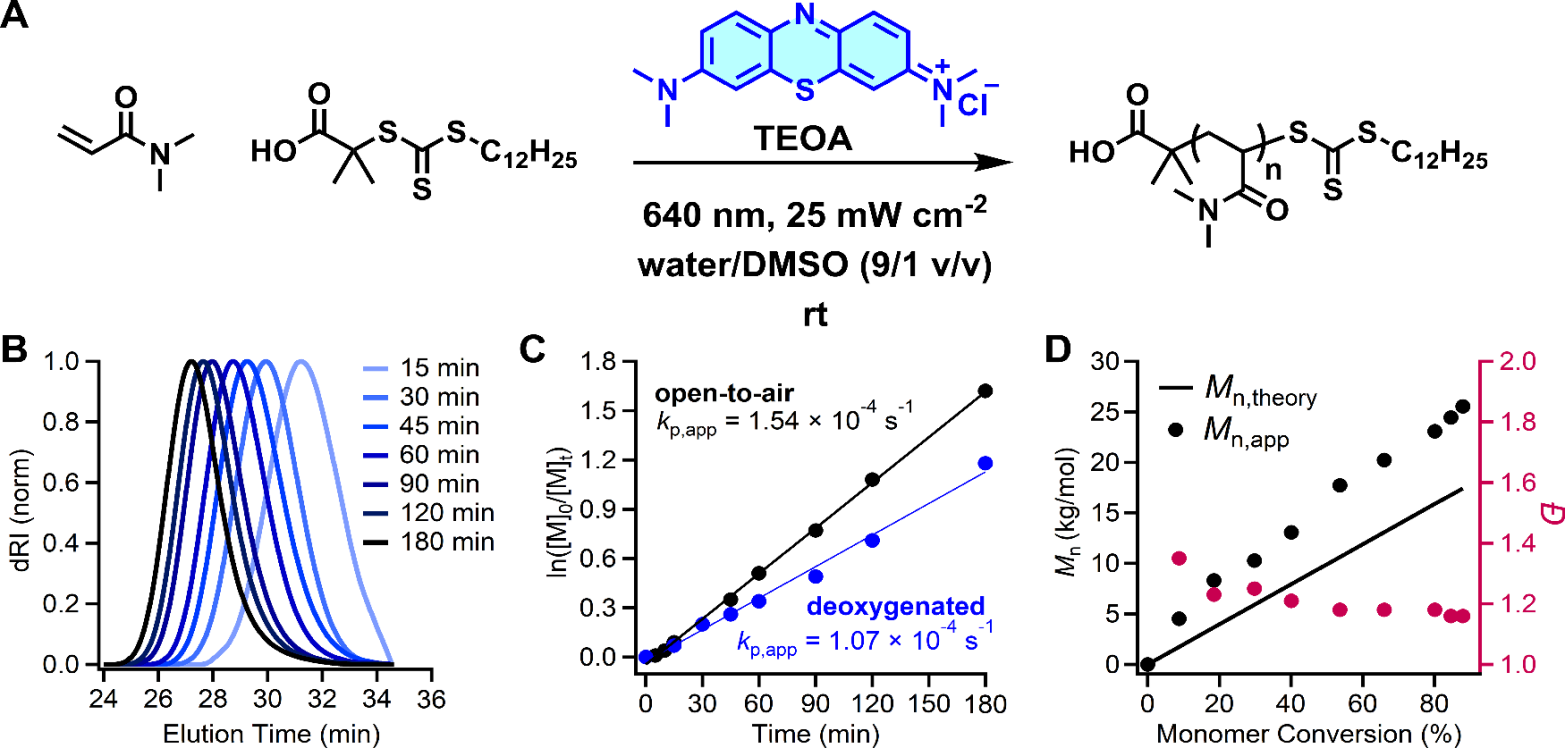
(A) Reaction scheme for the MB mediated RAFT polymerization of DMA. Reaction conditions: [DMA]/[DDMAT]/[TEOA]/[MB^+^] = 200/1.00/1.33/0.01; [DMA] = 3.0 M; [TEOA] = 20 mM; [MB^+^] = 150 μM. (B) Evolution of SEC traces during the MB^+^-mediated RAFT polymerization of DMA, demonstrating monomodal shifts toward lower elution times, indicative of increasing molecular weight. (C) Pseudo-first-order kinetic plot demonstrating linear kinetics and consistent radical flux under both open-to-air and deoxygenated conditions. Apparent rate constants: *k*_p,app_ = (1.54 ± 0.03) × 10–4 s^–1^ for open-to-air (R^2^ = 0.9984) and *k*_p,app_ = (1.07 ± 0.04) × 10–4 s^–1^ for deoxygenated (R^2^ = 0.9907). (D) Plot of *M*_n,app_ versus monomer conversion, showing a linear increase in molecular with relatively low dispersity (*Đ* ≤ 1.20) up to high monomer conversion (>90%). The observed discrepancy between *M*_n,theory_ and *M*_n,app_ is attributed to differences in hydrodynamic volume of PDMA in DMF relative to PMMA standards.

These results collectively demonstrated that the MB^+^/TEOA-mediated photoRAFT polymerization of DMA proceeds in a well-controlled manner.

### Varying Targeted Degrees of Polymerization

To evaluate the scope of molecular weight control, polymerizations targeting degrees of polymerization (*DP*_T_) from 50 to 20,000 were performed by varying the monomer-to-CTA ratio (Figure 3A and Table S3). At higher *DP*_T_ values (10,000–20,000), corresponding to significantly lower CTA concentrations, [MB^+^] had to be reduced to avoid excess radical generation relative to available CTA, which would otherwise compromise control. Remarkably, even at a *DP*_T_ of 20,000 with [MB^+^] = 26 μM (0.13 equiv relative to CTA), ^1^H NMR spectroscopy revealed a monomer conversion of 68%. Size exclusion chromatography with multiangle static light scattering (SEC-MALS) analysis showed an absolute number-average molar mass (*M*_n,abs_) of 1,290,000 g mol^–1^, closely matching the theoretical molar mass (*M*_n,theory_ = 1,350,000 g mol^–1^) with moderate dispersity (*Đ* = 1.52) (Table S3, entry 7). Increasing [MB^+^] to 50 or 100 μM under otherwise identical conditions led to lower-than-expected *M*_n,abs_ values and higher dispersities (Table S3, entries 8–9), consistent with reduced control at higher radical flux. Polymerizations performed under the same reaction conditions ([MB^+^] = 26 μM) but without a CTA resulted in significantly broader molecular weight distributions (*Đ* = 2.21), confirming the critical role of the CTA in enabling controlled synthesis of ultrahigh molecular weight (UHMW, > 1,000,000) polymers (Table S3, entry 10, and Figure S6).

**Figure 3:**
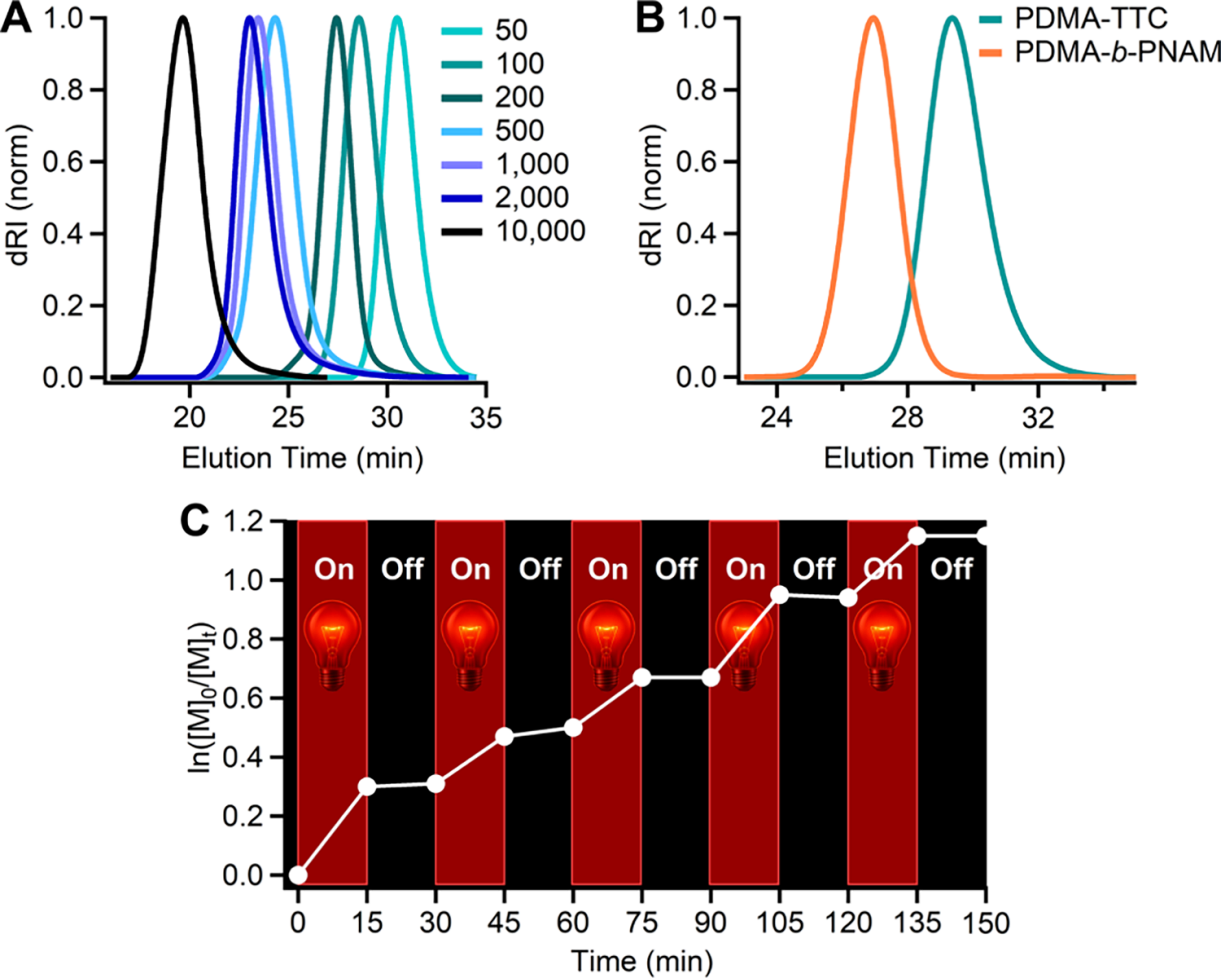
(A) SEC traces of PDMA with different targeted degrees of polymerization. (B) Chain extension of PDMA-TTC macroinitiator (*DP* = 100, *M*_n,theory_ = 9,650 g mol^–1^, *M*_n,app_ = 14,700 g mol^–1^, *Đ* = 1.14) with NAM, generating PDMA-*b*-PNAM block copolymer (*M*_n,theory_ = 30,900 g mol^–1^, *M*_n,app_ = 26,200 g mol^–1^, *Đ* = 1.16). (C) Temporal control of the MB^+^ RAFT polymerization of DMA. The 640 nm light source was switched on and off for 15 min each.

Since molecular weight fundamentally governs polymer physical properties and material performance,^72–74^ the ability to access UHMW polymers remains a longstanding synthetic goal.^75^ Polymers in this regime exhibit significantly enhanced chain entanglement, leading to exceptional tensile strength, toughness, and resistance to deformation compared to lower-molecular-weight analogues.^76–79^ Notably, UHMW polymers also offer opportunities to mimic the size, architecture, and viscoelastic behavior of natural macromolecules such as mucins and lubricins, which are critical for the design of next-generation soft materials.^80–83^ To address this challenge under ambient, oxygen-tolerant conditions, we explored an MB^+^/TEOA-mediated photoRAFT strategy. While this approach enables access to UHMW polyacrylamides, particularly when using high-propagation rate constants (*k*_p_) monomers such as acrylamides and employing water as a solvent to further enhance *k*_p_,^84–88^ it also presents limitations compared to alternative methods like photoiniferter polymerization,^60,89–91^ or high-pressure ATRP.^92,93^ At low MB^+^ concentrations (20–30 μM), monomer conversion typically plateaus around 60–70%, limiting the accessible molar mass to ∼ 1,500 kg mol^–1^. While increasing the MB^+^ concentration to 50–100 μM improves conversion, it simultaneously leads to reduced control due to the imbalance between radical generation and CTA concentration (Table S3, entries 7−9).

Nonetheless, the MB^+^/TEOA photoRAFT system offers a straightforward, metal-free, and oxygen-tolerant route to UHMW polyacrylamides with moderate dispersities (*Đ* ≈ 1.5). Unlike photoiniferter polymerization, which relies exclusively on the direct photolysis of the thiocarbonylthio compound (i.e., the CTA in RAFT) to generate radicals,^15^ the MB^+^/TEOA system—like thermal RAFT polymerization—depends on continuous radical generation from an exogenous source. This persistent radical flux increases the likelihood of termination events, particularly via diffusion-limited coupling between long polymer chains and low-molecular-weight radicals.^94–96^ Although termination slows at high chain lengths due to decreased translational diffusion,^97^ the continuous radical generation still imposes an upper limit on achievable molar mass. Thus, while offering less control and a more limited molar mass range than photoiniferter polymerization, the MB^+^/TEOA photoRAFT system provides a distinct practical advantage: oxygen tolerance under ambient conditions using low-energy red light.

### Chain-End Fidelity and Temporal Control

To confirm the ω-trithiocarbonate (TTC) chain-end fidelity of the polymers synthesized via MB^+^/TEOA photoRAFT polymerization, PDMA-TTC (*DP* = 100, *M*_n,theory_ = 9,650 g mol^–1^, *M*_n,app_ = 14,700 g mol^–1^, *Đ* = 1.14) was first synthesized and subsequently used as a macroinitiator for chain extension with *N*-acryloylmorpholine (NAM), resulting in the formation of a PDMA-*b*-PNAM block copolymer. SEC analysis revealed a clean shift to lower elution times, indicating an increase in molecular weight of the resulting block copolymer (*M*_n,app_ = 26,200 g mol^–1^, *Đ* = 1.16, *M*_n,theory_ = 30,900 g mol^–1^), with no evidence of tailing or a low-molecular-weight shoulder that would suggest the presence of unreacted macroinitiator—supporting efficient chain extension. (Figure 3B). In addition, ^1^H NMR spectroscopy of the purified block copolymer demonstrated the presence of the NAM block (Figure S4). End-group analysis by ^1^H NMR spectroscopy was further conducted on a low molar mass PDMA-TTC sample (*DP* = 45, *M*_n,app_ = 6,580 g mol^–1^, *Đ* = 1.17, Table S3, entry 1) to assess chain-end fidelity. By comparing the relative integrals of the methine proton (δ = 5.20 ppm, 1H) at the terminal repeat unit α to the TTC moiety and the methyl protons (δ = 0.87 ppm, 3H) of the dodecyl group, complete chain-end retention was confirmed (Figure S5). Gratifyingly, chain-extension of a UHMW PDMA-TTC macroinitiator (*M*_n,abs_ = 1,190 kg mol^–1^, *Đ* = 1.38) with NAM under similar conditions resulted in a UHMW PDMA-*b*-PNAM block copolymer (*M*_n,abs_ = 1,690 kg mol^–1^, *Đ* = 1.34) with a clear SEC shift with minimal tailing, demonstrating preserved chain-end fidelity even at very high degrees of polymerization (Figure S7B). Furthermore, ^1^H NMR spectroscopy showed the characteristic signals of the NAM block (Figure S8). These results highlight the robustness of the MB^+^/TEOA photoRAFT system for the synthesis of UHMW block copolymers under ambient, oxygen-tolerant conditions.

To further evaluate the extent of irreversible termination in the MB^+^/TEOA-mediated photoRAFT polymerization, we estimated the concentration of terminated (or initiated) chains using established kinetic models.^98^ Using reported propagation rate constant for DMA in water at 25 °C (*k*_p_ = 4.35 × 10^4^ L mol^–1^ s^–1^)^99^ and a termination rate constant (*k*_t_ = 3.8 × 10^7^ L mol^–1^ s^–1^),^100^ and taking into account the reaction time and monomer conversion under our experimental conditions, we calculated that the concentration of terminated chains was only ∼ 1.5% relative to the initial CTA concentration (see Supporting Information). This low level of termination is consistent with the high molar masses, and excellent end-group fidelity demonstrated by efficient chain extension, collectively supporting the controlled nature of the polymerization.

To assess the temporal control of the MB^+^/TEOA-mediated photoRAFT polymerization, an on/off light experiment was performed using DMA under optimized conditions (Figure 3C). The polymerization could be effectively paused and resumed by switching the red-light source on and off. During dark periods, monomer conversion remained negligible, indicating no polymerization in the absence of light. Upon reillumination, the polymerization resumed without delay, confirming the living nature of the propagating chains and excellent responsiveness of the system. Five repeated on/off cycles had no observable impact on the kinetics or control, underscoring the robustness of the MB^+^/TEOA system and its potential for spatiotemporal regulation under ambient conditions.

### Polymerization under Different Light Wavelengths

While red-light irradiation is advantageous for bioconjugation and offers deeper penetration into biological media, the broad absorption spectrum of MB^+^ enables polymerization under a wide range of wavelengths, spanning from long-wave UV to NIR light. This spectral versatility not only allows tuning of the activation wavelength but also facilitates integration with orthogonal photochemical processes,^101,102^ such as photocleavable protecting groups,^103^ or light-sensitive biomolecules,^104^ without mutual interference. As a result, the system is well-suited for applications involving multiwavelength control or light-sensitive conjugates. Except for 370 nm UV light, which resulted in a significant discrepancy between theoretical and measured molecular weights (Table 2, entry 1, Figure S3), polymerizations conducted under blue, green, red, and NIR light achieved high conversions (>90%) and low dispersities (*Đ* < 1.14) (Table 2, entries 2–6, Figure S3), demonstrating the robustness and wavelength tolerance of the MB^+^/TEOA-mediated photoRAFT system. Notably, in the absence of MB^+^, blue-light irradiation still led to 50% conversion, consistent with direct photolysis of the thiocarbonylthio moiety via a photoiniferter mechanism (Table 2, entry 3).^105,106^

**Table 2:** Polymerization of DMA Using Different Light Wavelengths^a^

Entry	Light	λ_max_(nm)	Intensity(mW cm^–2^)	[MB^+^](μM)	Conv.(%)^b^	*M*_n,theory_(kg mol^–1^) *^c^*	*M*_n,app_(kg mol^–1^) *^d^*	*Đ* ^d^
1	UV	370	7.0	150	>99	19.8	40.1	1.17
2	blue	456	30	150	>99	19.8	27.4	1.11
3	blue	456	30	0	50	9.94	14.0	1.16
4	green	525	12	150	96	18.9	26.6	1.11
5	red	640	25	150	90	17.8	22.4	1.14
6	NIR	740	20	150	96	18.9	25.9	1.08
7	sunlight	-	-	50	95	18.8	23.1	1.11
8^e^	sunlight	-	-	50	94	18.6	23.4	1.11

a Reaction conditions: [DMA]/[DDMAT]/[MB^+^]/[TEOA] = 200/1/x/1.33, [DMA] = 3.0 M, [TEOA] = 20 mM, in water with DMSO (10% v/v), irradiated for 10 h under different LEDs or 1 h under sunlight.

b Monomer conversion determined by ^1^H NMR spectroscopy.

c Theoretical number-average molar masses (*M*_n,theory_) were determined from the monomer conversion from ^1^H NMR spectroscopy.

d Apparent number-average molar masses (*M*_n,app_) and dispersity (Đ) were determined by SEC using DMF + 50 mM LiBr relative to PMMA standards.

e This polymerization was done on 13 mL scale corresponding to 4.0 mL DMA.

### Polymerization Driven by Sunlight

Given the broad absorption spectrum of MB^+^ and the accessibility of sunlight, we explored whether the MB^+^-mediated RAFT polymerization could proceed using direct sunlight under open-to-air, unstirred conditions (Figure 4A & 4B). Table S4 (Supporting Information) lists the measured sunlight intensities at wavelengths corresponding to the LED sources used in this study. Two reaction volumes were tested: a small-scale setup (3.3 mL) and a larger-scale setup (13 mL). In both cases, polymerization proceeded rapidly at [MB^+^] = 50 μM, reaching >94% conversion within 1 h. The resulting polymers displayed nearly identical molecular weights and dispersities (Table 2, entries 7 and 8, Figure S3B), comparable to those obtained under controlled LED irradiation. Kinetic analysis further confirmed well-controlled polymerization, with an apparent propagation rate constant (*k*_p,app_ = 7.78 × 10^–4^ s^–1^) exceeding that observed under red-light conditions (*k*_p,app_ = 1.54 × 10^–4^ s^–1^) (Figure 4C–E).

**Figure 4:**
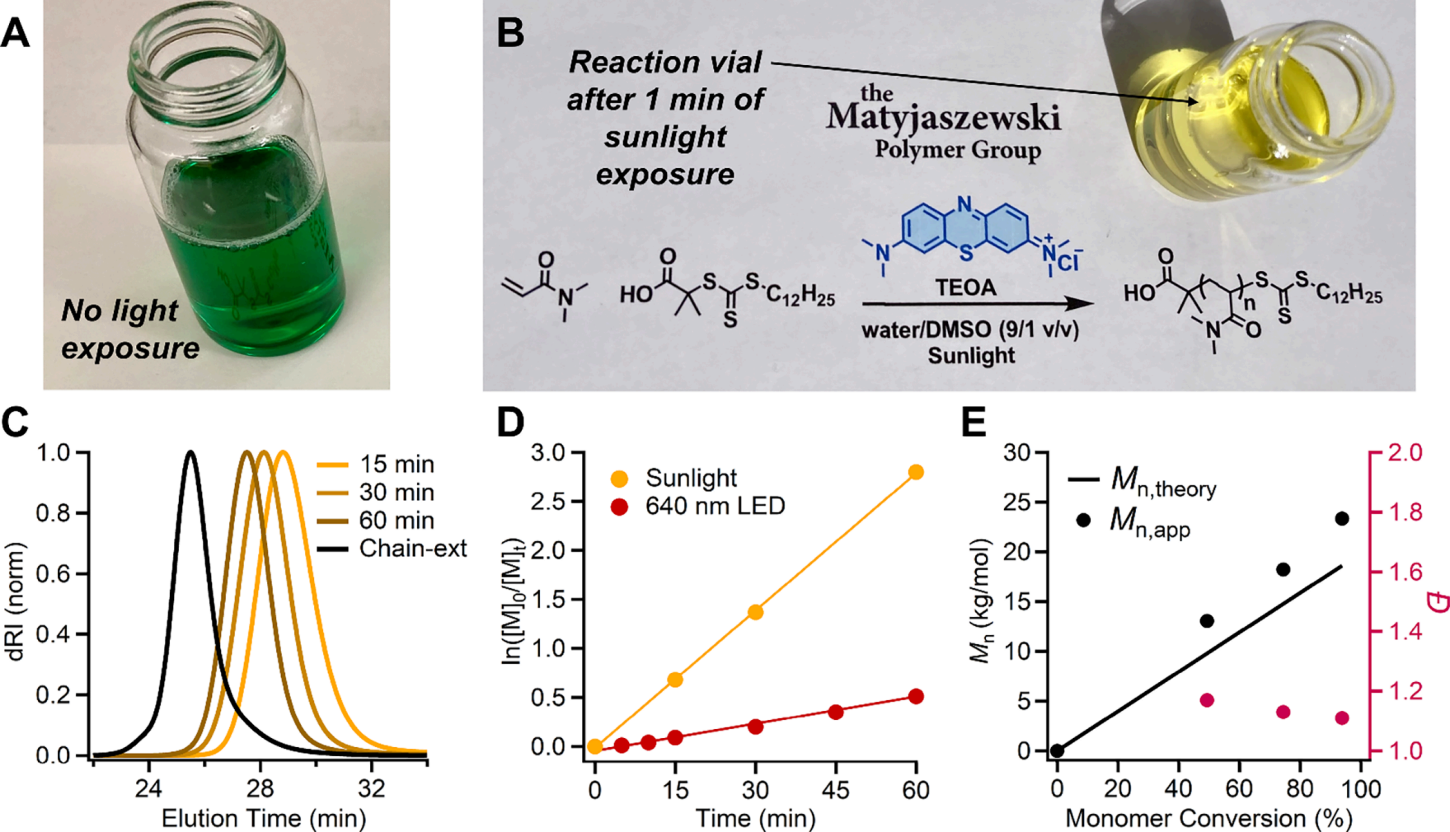
Sunlight-driven MB^+^/TEOA-mediated photoRAFT polymerization of DMA. (A) Reaction mixture (total volume = 13 mL, containing 4.0 mL DMA) prior to sunlight exposure, showing a green color resulting from the combined absorption of blue-colored MB^+^ and yellow-colored TTC CTA. The reaction was conducted fully open to air and without stirring. (B) Reaction scheme and photograph of the same reaction mixture after just 1 min of sunlight exposure, during which the green color rapidly faded due to the formation of colorless leucomethylene blue (LMB), confirming efficient photoactivation. Reaction conditions: [DMA]/[DDMAT]/[TEOA]/[MB^+^] = 200/1.00/3.33/0.003; [DMA] = 3.0 M; [MB^+^] = 50 μM; [TEOA] = 50 mM in phosphate-buffered saline (PBS, pH = 8.0, 100 mM)/DMSO (9:1 v/v). The temperature measured by the reaction vessel was 50 °C. (C) Evolution of SEC traces over time during the sunlight-driven polymerization, showing a clean, monomodal shift toward lower elution times indicative of increasing molecular weight. Chain extension with NAM was subsequently performed in situ without prior isolation or purification of the PDMA-TTC macroinitiator. The black trace shows successful chain extension, confirming high end-group fidelity. (D) Pseudo-first-order kinetic plot demonstrating linear kinetics and consistent radical flux. Apparent rate constants: *k*_p,app_ = (7.78 ± 0.07) × 10–4 s^–1^ for sunlight (R^2^ = 0.9995), and *k*_p,app_ = (1.54 ± 0.03) × 10–4 s^–1^ for 640 nm red light (R^2^ = 0.9857). (E) Plot of *M*_n,app_ versus monomer conversion, showing a linear increase in molecular mass with relatively low dispersity (*Đ* ≤ 1.20) up to high monomer conversion (>95%).

To assess the contribution of the photoiniferter pathway, the 3.3 mL sunlight experiment was repeated without MB^+^ under otherwise identical conditions. Conversion after 1 h was only 9.3% versus 94% with MB^+^, highlighting the essential role of MB^+^ in overcoming oxygen inhibition. Once oxygen tolerance is established, a larger contribution from the intrinsic photoiniferter process under sunlight is likely.

Exposure to high-energy, short-wavelength UV components in sunlight can raise concerns about potential TTC end-group decomposition,^107^ which would compromise chain-end fidelity and polymerization control. To assess this, we performed a chain-extension experiment using the crude PDMA (13 mL scale, Figure 4B) directly as a macroinitiator for NAM polymerization, without prior isolation or purification. NAM and MB^+^ were added directly to the reaction mixture, followed by sunlight exposure. ^1^H NMR spectroscopy showed quantitative monomer conversion after 2 h, and SEC analysis revealed a clean shift to lower elution volume with no detectable residual PDMA macroinitiator. These results confirm excellent TTC end-group retention and chain-end fidelity under sunlight irradiation (Figure 4C).

### Monomer Scope

We next evaluated the monomer scope of the MB^+^/TEOA-mediated photoRAFT polymerization by exploring a diverse range of hydrophilic monomers, including acrylamides, methacrylamides, acrylates, and methacrylates bearing neutral, charged, or zwitterionic functionalities (Figure 5, Table S5) All polymerizations were performed targeting a *DP* of 200 under red-light irradiation using appropriate CTAs tailored to each monomer class. For acrylamido and acrylic monomers, trithiocarbonates such as DDMAT and CETPA were employed, while for methacrylamides and methacrylates, TTC-based CTAs 4-cyano-4-(((dodecylthio)­carbonothioyl)­thio)­pentanoic acid (CDP) and 4-((((2-carboxyethyl)­thio)­carbonothioyl)­thio)-4-cyanopentanoic (CETCPA) and the dithiobenzoate-based CTA 4-cyano-4-(phenyl­carbono­thioyl­thio)­pentanoic acid (CPADB) were used. In all cases, polymers were obtained with moderate to high monomer conversions, molecular weights in good agreement with theoretical values, and narrow molecular weight distributions (*Đ* < 1.30), highlighting the versatility and robustness of this oxygen-tolerant system (Table S5).

**Figure 5:**
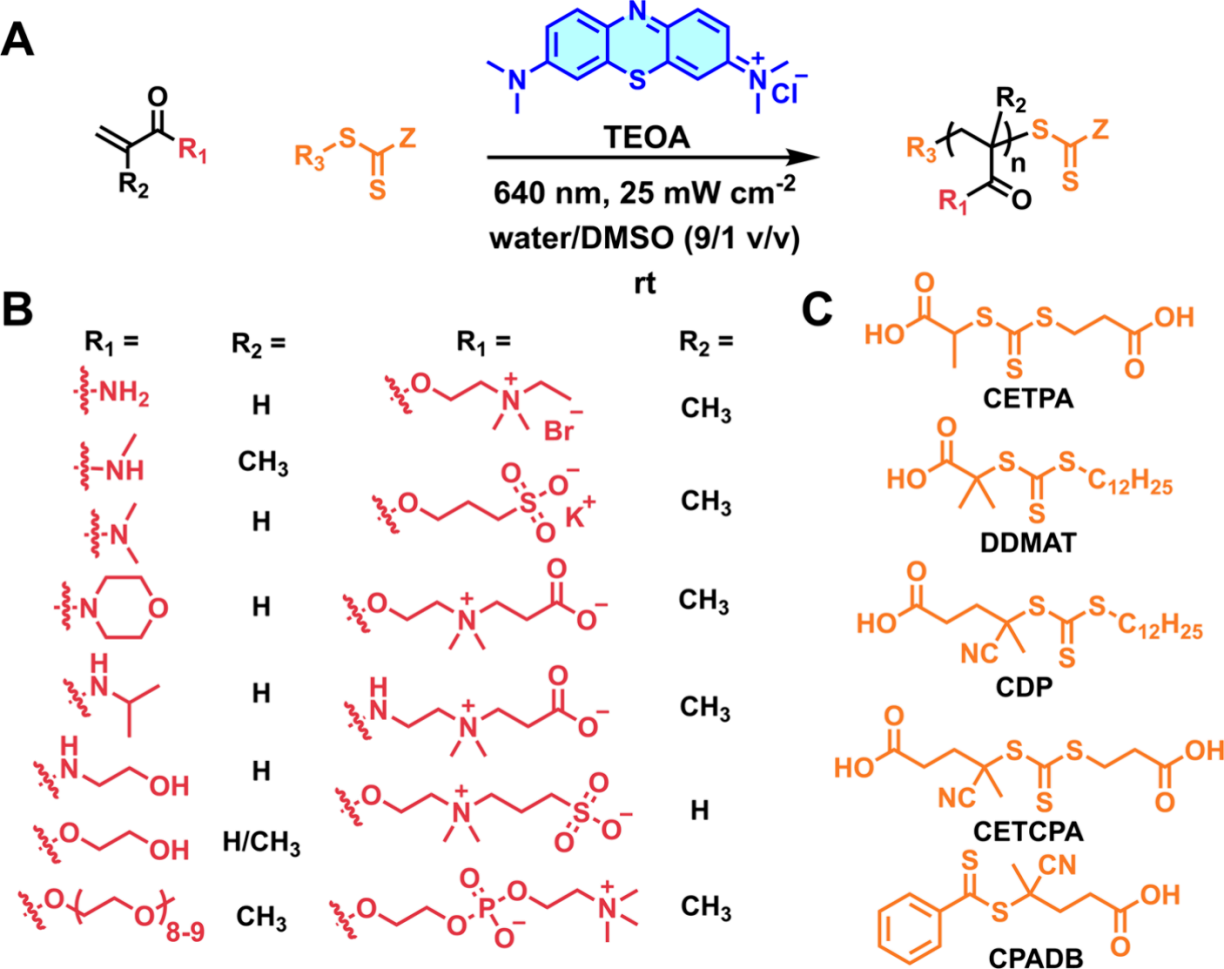
(A) Reaction scheme for the red light-driven photoRAFT polymerization of various (meth-)­acrylates and (meth-)­acrylamides using MB^+^/TEOA photosensitizing system. (B) Monomer scope demonstrating the compatibility of the MB^+^-mediated system with (meth)­acrylates and (meth-)­acrylamides bearing polar, charged, and zwitterionic functionalities. (C) CTA scope.

Notably, the polymerization of methacrylamides remains relatively underexplored via RDRP techniques. To address this, we investigated the polymerization of *N*-methylmethacrylamide (NMMA) and *N*-(2-hydroxypropyl)­methacrylamide (HPMA) using the MB^+^-mediated photoRAFT system (Table S5). For NMMA, all tested CTAs in water/DMSO (9:1 v/v) resulted in low conversions (<20%). Replacing water with ethylene glycol markedly increased conversion, with the best control obtained using CETCPA, yielding excellent agreement between theoretical and measured molar masses and low dispersity (*M*_n,theory_ = 7,870 g mol^–1^, *M*_n,app_ = 8,360 g mol^–1^, *Đ* = 1.15, Table S5, entry 6). Similarly, HPMA polymerized efficiently in EG/DMSO (9:1 v/v) using CDP, producing molar masses in good agreement with theoretical values and low dispersity (*M*_n,theory_ = 17,000 g mol^–1^, *M*_n,app_ = 17,800 g mol^–1^, *Đ* = 1.23, Table S5, entry 7). Detailed summary of the optimization of the polymerization of methacrylamide monomers, HPMA and NMMA is summarized in Table S6 (Supporting Information).

Having already demonstrated access to UHMW PDMA (*M*_n,abs_ > 1,000,000 g mol^–1^), we next explored the compatibility of other monomers with MB^+^/TEOA-mediated photoRAFT polymerization at high targeted *DP*s (*DP*_T_ = 10,000–20,000).

Polymerizations were carried out with 2-(2-carboxyethylsulfanylthiocarbonylsulfanyl)-2-methylpropionic acid (CEPTA) as the CTA for acrylamide and acrylate monomers, and CDP for methacrylate monomers. Using CEPTA, various acrylamides—including *N*-acryloylmorpholine (NAM), acrylamide (Am), and *N*-hydroxyethyl acrylamide (HEAm)—as well as the acrylic monomer 2-(2-(2-methoxyethoxy)­ethoxy)­ethyl acrylate (TEGA), were successfully polymerized to *M*_n,abs_ values up to 1,300 kg mol^–1^. (Figure S12, Table S7, entries 1–5). This approach was further extended to charged monomers, including the cationic quaternary ammonium-containing acrylate (DMEQAA) and methacrylate (DMEQAMA), the anionic sulfopropyl methacrylate (SPMA), and the zwitterionic methacrylates carboxybetaine methacrylate (CBMA) and sulfobetaine methacrylate (SBMA) (Table S7, entries 6–10). Across all monomer classes, highly water-soluble UHMW polyelectrolytes and polyzwitterions were obtained with *M*_n,abs_ values up to 2,700 kg mol^–1^ and moderate dispersities, as determined by aqueous SEC-MALS. These results underscore the broad monomer scope of the MB^+^/TEOA system and its ability to produce UHMW polymers with diverse chemical and charge characteristics under mild, oxygen-tolerant conditions.

### Proposed Mechanism

The MB^+^/TEOA-mediated photoRAFT polymerization likely proceeds via a photoinitiated radical pathway, rather than direct photoinduced electron/energy transfer (PET) process involving the thiocarbonylthio-based CTA. Upon visible-light excitation, MB^+^ is promoted from its ground state to the singlet excited state (^1^MB^+^*), which rapidly undergoes intersystem crossing (ISC) to generate the longer-lived triplet excited state (^3^MB^+^*).^108^ This photoexcited species is reductively quenched by tertiary amines such as TEOA or DIPEA, forming a short-lived excited-state charge-transfer complex (exciplex) that collapses via electron transfer to generate the semireduced MB radical (MB•) and an amine radical cation (R_3_N^+^•).^69,109^ The latter undergoes α-C–H deprotonation to yield an α-aminoalkyl radical, which is sufficiently reactive to initiate polymerization by adding to electron-deficient vinylic monomers, forming propagating chains.^110–112^

MB• can follow two competing pathways: (1) reoxidation by molecular oxygen to regenerate MB^+^, enabling redox cycling; or (2) hydrogen atom transfer (HAT) from excess amine or solvent to form LMB, the fully reduced, colorless, and photochemically inactive form of MB^+^.^113^ While LMB can in principle be reoxidized by oxygen to regenerate MB^+^, this process is slow and may not fully prevent the accumulation of photoinactive species under ambient conditions. In contrast, MB^+^/Cu dual-catalyzed photoATRP systems benefit from rapid and efficient reoxidation of MB• by Cu­(II) complexes (*E*_1/2_(Cu^2+^/Cu^+^) = −0,23 V vs SCE), thereby avoiding photobleaching and enabling sustained catalytic activity under prolonged irradiation.^51,52^ This reoxidation pathway is absent in the metal-free MB^+^/TEOA RAFT system, leading to gradual MB^+^ photobleaching over time.

This mechanistic pathway is consistent with redox potentials: MB^+^ in its triplet excited state (^3^MB^+^*) has an excited-state reduction potential of *E*_1/2_(^3^MB^+^*/MB^•^) = +1.60 V vs SCE, allowing thermodynamically favorable reduction by amines such as TEOA (*E*_1/2_(TEOA^+^•/TEOA) = +0.76 V vs SCE).^114,115^ Conversely, the excited-state oxidation potential of ^3^MB^+^* (*E*_1/2_(^3^MB^+^*/MB^2+^) = – 0.68 V vs SCE) is insufficient to reduce TTC-based CTAs like DDMAT (*E*_1/2_(DDMAT/DDMAT•^–^) = – 1.56 V vs SCE in MeCN).^116,117^ Thus, direct PET activation of the RAFT agent by MB^+^ is thermodynamically inaccessible. Supporting this conclusion, Boyer and co-workers observed no polymerization of methyl methacrylate using MB^+^ and CPADB under red light in the absence of a tertiary amine, further demonstrating that MB^+^ cannot activate the RAFT agent via electron transfer alone.^118^ This distinction is critical, as MB^+^ lacks both the redox potential and any demonstrated ability to engage in energy transfer with thiocarbonylthio moieties. Therefore, unlike PET-RAFT that relies on direct photoactivation of the RAFT agent via energy or electron transfer, the MB^+^/TEOA system operates through external radical generation via α-aminoalkyl radicals derived from amine oxidation. These radicals then initiate polymerization, while the RAFT agent governs propagation and termination via conventional RAFT equilibrium steps (Figure 6B).

**Figure 6:**
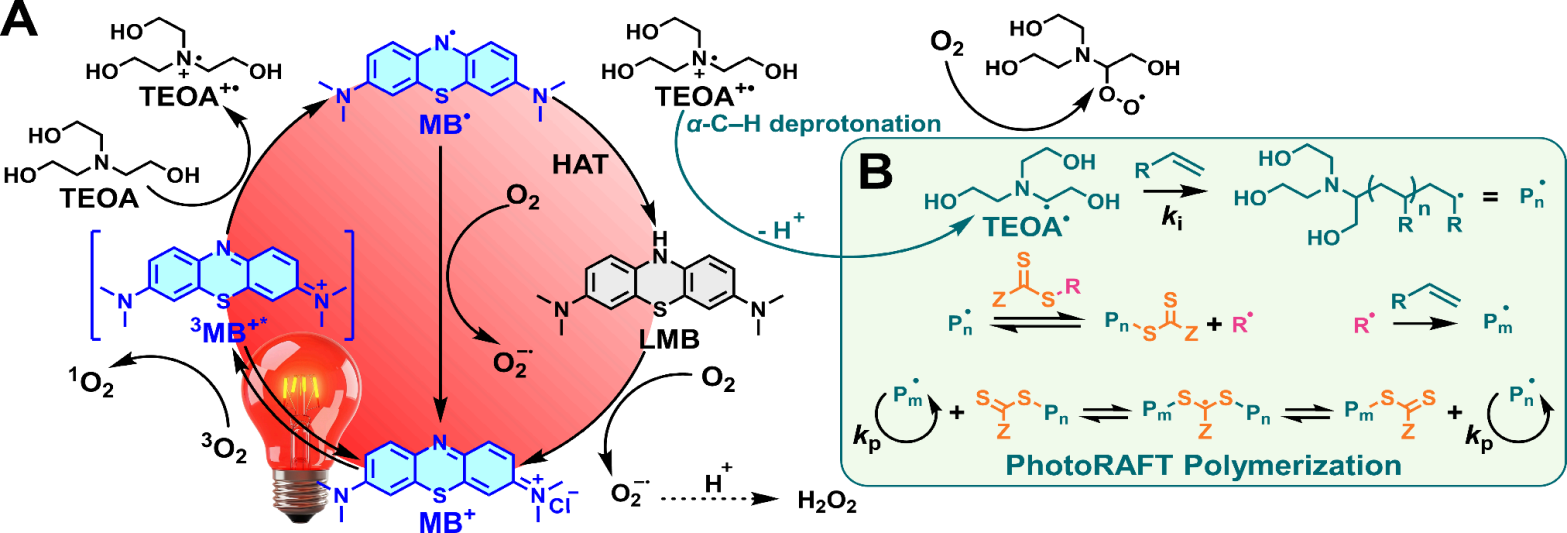
(A) Proposed mechanism for MB^+^/TEOA-mediated photoRAFT polymerization. Upon visible-light excitation, MB^+^ transitions to its triplet excited state (^3^MB^+^*), which undergoes reductive quenching by tertiary amines (e.g., TEOA) to generate the semireduced MB radical (MB•) and an amine radical cation (R_3_N^+^•). Subsequent α-C–H deprotonation yields highly reactive α-aminoalkyl radicals capable of initiating polymerization. Competing pathways for MB• include reoxidation by O_2_ to regenerate MB^+^ or hydrogen atom transfer (HAT) to form photoinactive leucomethylene blue (LMB). (B) RAFT polymerization is initiated by α-aminoalkyl radical addition to electron-deficient vinyl monomers, forming propagating polymer radicals (P_n_^•^). These radicals subsequently add to CTA, forming a thiocarbonylthio (TCT) intermediate radical, which fragments to release the R group (R^•^), a second initiating radical that can propagate to form a new polymer radical (P_m_^•^). Both propagating radicals (P_n_^•^ and P_m_^•^) enter a reversible degenerative chain transfer process characteristic of the RAFT equilibrium, enabling controlled polymerization through dynamic exchange between active and dormant species.

The MB^+^/TEOA system displays inherent oxygen tolerance because continuously generated α-aminoalkyl radicals rapidly react with dissolved oxygen, depleting it from the medium, after which they can initiate polymerization with electron-deficient monomers.^111^ Additionally, ^3^MB^+^* can be directly quenched by oxygen to form singlet oxygen (^1^O_2_),^119^ and MB• can reduce oxygen to the superoxide radical anion (O_2_•^–^),^120^ further accelerating deoxygenation. MB• may also undergo HAT to form LMB, which is photochemically inactive. Although LMB can be reoxidized to MB^+^ by oxygen,^121^ this regeneration becomes inefficient once oxygen is depleted. In contrast, MB^+^/Cu dual-catalyzed photo-ATRP maintain an active catalyst pool through Cu­(II)-mediated reoxidation of MB•,^122^ preventing LMB accumulation. The absence of this secondary oxidant in the RAFT system renders it more susceptible to irreversible photobleaching over time. Notably, while superoxide radical anion can form hydrogen peroxide (favored under acidic conditions), which may promote hydroxyl radical formation via Fenton-type reactions in Cu-containing systems, this is not a concern under metal-free RAFT conditions.

Overall, this photoRAFT approach represents a mechanistically distinct, oxygen-tolerant alternative to PET-RAFT, relying on broad-spectrum excitation of MB^+^ and amine-derived radical generation to enable controlled polymerizations under mild, open-air conditions.

## Conclusions

In summary, we report a red-light-driven, methylene blue (MB^+^)/TEOA-mediated RAFT polymerization system that operates under fully ambient, open-air conditions—including in direct sunlight—without requiring deoxygenation. This platform enables controlled polymerization of a broad range of vinylic monomers, including acrylates, methacrylates, acrylamides, and methacrylamides, bearing polar, charged, and zwitterionic functionalities. High monomer conversions and low to moderate dispersities are achieved across a wide range of reaction conditions, wavelengths, and scales, with excellent end-group fidelity and robust kinetic control.

Notably, the method enables access to ultrahigh molecular weight (UHMW) polymers (*M*_n,abs_ > 1,000,000 g mol^–1^) under mild, metal-free, and scalable conditions—an outcome rarely achieved in oxygen-tolerant RDRP systems. While the accessible molar mass range and control are more limited than that of photoiniferter polymerization, the complete oxygen tolerance, low-energy and biocompatible red and NIR-light activation, and operational simplicity of this MB^+^/TEOA-mediated photoRAFT system offer key advantages. Collectively, these attributes make it a powerful addition to the RDRP toolbox, with particular relevance for applications in biomaterials, high-throughput screening, surface functionalization, and polymer–protein conjugation, where mild, metal-free conditions are critical.

## Supplementary Material



## Notes

Materials, instrumentation, synthesis, supplementary figures (PDF)
