# Supplementary material for: Red Light-Driven, Oxygen-Tolerant RAFT Polymerization Enabled by Methylene Blue
Source: J Am Chem Soc. 2025 Aug 21;147(35):32096–109. doi: 10.1021/jacs.5c10541 (PMC12412174; doi:10.1021/jacs.5c10541)
Supplement: Supplementary file 1 [file ja5c10541_si_001.pdf]

Supporting Information for

Red-Light-Driven, Oxygen-Tolerant RAFT Polymerization Enabled by Methylene Blue

Lucca Trachsel, Ivan O. Levkovsky, Xiaolei Hu, Hironobu Murata, Krzysztof Matyjaszewski*

Department of Chemistry, Carnegie Mellon University, Pittsburgh, Pennsylvania 15213, USA

E-mail: km3b@andrew.cmu.edu

Supporting Information:

MATERIALS	2
INSTRUMENTATION	3
SYNTHESIS	4
SUPPLEMENTARY FIGURES	13
REFERENCES	20

Materials

All reagents and solvents were purchased at the highest commercial grade and used as received unless otherwise noted.

The following monomers were obtained from MilliporeSigma: 2-methacryloyloxyethyl phosphorylcholine (MPC, 97%, stabilized with ≤100 ppm monomethyl ether hydroquinone (MEHQ)), 3-sulfopropyl methacrylate potassium salt (SPMA, 98%), 3-((2-(acryloyloxy)ethyl)dimethylammonio)propane-1-sulfonate (sulfobetaine acrylate, SBA, 98%), acrylamide (Am, ≥99%), *N*-isopropylacrylamide (NIPAM, 97%, recrystallized from hexanes and dried under high vacuum), *N*,*N*-dimethylacrylamide (DMA, 99%, contains 500 ppm MEHQ), 4-acryloylmorpholine (NAM, 97%, contains 1,000 ppm MEHQ), *N*-hydroxyethylacrylamide (HEAm, 97%, contains 1,000 ppm MEHQ), *N*-methylmethacrylamide (NMMA, 98%, stabilized with hydroquinone), 2-hydroxyethyl acrylate (HEA, 96%, contains 200–650 ppm MEHQ), 2-hydroxyethyl methacrylate (HEMA, ≥99%, contains ≤50 ppm MEHQ), oligo(ethylene glycol) methyl ether methacrylate (M_n = 500 g/mol, $OEGMA_{500}$, 900 ppm MEHQ). 3-((2-(Methacryloyloxy)ethyl)dimethylammonio)propanoate (carboxybetaine methacrylate, CBMA, >98.0%) and 2-(2-(2-methoxyethoxy)ethoxy)ethyl acrylate (TEGA, >90.0%, stabilized with MEHQ) were purchased from Tokyo Chemical Industry. All liquid monomers were passed through a plug of basic alumina to remove inhibitors and acidic impurities and were stored at –20 °C.

4-Cyano-4-(phenylcarbonothioylthio)pentanoic acid (CPADB, 97%) was purchased from Strem Chemicals. 2,2'-(Carbonothioyldisulfanediyl)bis(2-methylpropanoic acid) (CMP, 97%), 3-((((1-carboxyethyl)thio)carbonothioyl)thio)propanoic acid (CETPA, 95%), and 4-((((2-carboxyethyl)thio)carbonothioyl)thio)-4-cyanopentanoic acid (CETCPA, 95%) were purchased from Boron Molecular and used as received.

2-(Dodecylthiocarbonothioylthio)-2-methylpropanoic acid (DDMAT),[1] 4-cyano-4-(((dodecylthio)carbonothioyl)thio)pentanoic acid (CDP)[2], 2-(2-carboxyethylsulfanylthiocarbonylsulfanyl)-2-methylpropionic acid (CEPTA)[3], and 2-(dimethylethylammonio)ethyl methacrylate bromide (DMEAEMA)[4] were synthesized as reported previously.

Deuterated chloroform ($CDCl_3$), deuterated dimethylsulfoxide (DMSO-d_6) and deuterium oxide (D_2O) were obtained from Cambridge Isotope Laboratories.

Instrumentation

Photoreactor for MB⁺/TEOA photoRAFT polymerization. Polymerizations were conducted in a EvoluChem™ PhotoRedOx Box (HepatoChem) using various LEDs. PR160L-type LEDs (Kessil) were used for UV (370 nm, 7.0 mW cm^{-2}), blue (456 nm, 30 mW cm^{-2}), green (525 nm, 12 mW cm^{-2}), and red (640 nm, 25 mW cm^{-2}) light. Near-infrared (NIR, 740 nm, 20 mW cm^{-2}) LEDs were purchased from HepatoChem. Light intensities were measured at the location of the reaction vessels using a PM100D optical power meter (Thorlabs).

Nuclear Magnetic Resonance (NMR) Spectroscopy. ^1H NMR spectra were collected using a Bruker Advance 500 MHz NMR spectrometer. $CDCl_3$, DMSO-d_6 and D_2O were used as solvents, and the residual solvent signal served as a reference. Monomer conversion was determined using ^1H NMR spectroscopy performed on a Magritek Spinsolve Ultra 60 MHz benchtop spectrometer using DMF or 1,3,5-trioxane as an internal standard.

Size-Exclusion Chromatography (SEC). The apparent number-average molar masses ($M_{n,app}$) and dispersity ($Đ$) of polymers soluble in *N*,*N*-dimethylformamide (DMF) were measured relative to poly(methyl methacrylate) (PMMA) standards using an Agilent 1260 Infinity II isocratic pump, a column set containing 3 PSS analytical columns (GRAM Lux 10^2, 10^3, 10^4, 10^5 Å pore sizes), and Agilent 1260 Infinity II refractive index (RI) detector. The eluent was DMF + 50 mM LiBr, operated at 50 °C with a flow rate of 1 mL min^{-1}. Absolute number-average molar masses ($M_{n,abs}$) were determined using the same setup with the addition of a PSS SLD2020 multi-angle light scattering (MALS) detector. $M_{n,abs}$ and $Đ$ values were calculated using using refractive index increment (dn/dc) values determined using 100% mass recovery method.

Aqueous SEC-MALS characterizations for polymers not soluble in DMF including PAm, PCBMA, PMPC, PSBA, PSPMA, PDMEAEMA were performed using Agilent SEC system (1260 Infinity II) equipped with UV detector, DAWN HELEOS-II (Wyatt) MALS detector and Optilab T-rEX (Wyatt Technology) RI detector. Measurements of PCBMA, PMPC, PSBA, PAm homopolymers were performed using a SUPREMA Lux 3000Å, 8 × 300 mm, 10 μm and guard column (PSS) at a flow rate of 0.5 mL min^{-1} with Dulbecco's Phosphate Buffered Saline and 0.02wt% sodium azide as eluent. Measurement of anionic PSPMA homopolymer was performed using a using a SUPREMA Lux 3000Å, 8 × 300 mm, 10 μm and guard column at a flow rate of 0.5 mL min^{-1} with 10 mM sodium phosphate (pH 8.5) and 100 mM sodium chloride as eluent. Measurement of cationic PDMEAEMA homopolymer was performed using a NOVEMA Max Lux 3000Å, 8 × 300 mm, 10 μm and guard column (PSS) at a flow rate of 0.5 mL min^{-1} with the mixture of 10 mM sodium phosphate (pH 4.5) and 100 mM sodium chloride as eluent.

UV-Vis Spectroscopy. UV-Vis absorption spectra of MB^+ were recorded on an Agilent Cary 5000 UV-Vis-NIR spectrophotometer in the range of 200-800 nm with 1.0 nm resolution and 600 nm min^{-1} scan rate.

Synthesis

General procedure for MB^+/TEOA photoRAFT polymerization of DMA under red light irradiation.
Stock solutions of MB^+ (3.13 mM in H_2O), DDMAT (424 mM in DMSO), TEOA (583 mM in DMSO) were first prepared. A typical polymerization reaction (1.62 mL total volume) was prepared in a 1-dram glass vial by combining the following components under protection from ambient light: DMA (500 µL, 4.85 mmol, 200 equiv), DMF (100 µL, internal standard for ^1H NMR spectroscopy), DDMAT stock (57 µL, 24 µmol, 1.0 equiv), TEOA stock (55 µL, 32 µmol, 1.3 equiv), MB^+ stock (78 µL, 0.24 µmol, 0.1 equiv), H_2O (0.83 mL). DMSO content was adjusted to ensure a final H_2O/DMSO ratio of 9/1 (v/v).
Final reaction conditions: [DMA]/[DDMAT]/[MB^+]/[TEOA] = 200/1.00/0.01/1.33, [DMA] = 3.0 M, [MB^+] = 150 µM, [TEOA] = 20 mM. Prior to irradiation, a 50 µL aliquot was mixed with 550 µL D_2O to determine the initial monomer conversion (t = 0) by ^1H NMR spectroscopy. Monomer conversion was calculated from the decrease in the vinylic proton signal (1H, δ = 6.69 ppm) relative to DMF (1H, δ = 7.92 ppm) used as an internal standard. The reaction mixture was then irradiated with red LEDs (640 nm, 25 mW cm^{-2}), and aliquots were taken at defined timepoints for analysis by ^1H NMR spectroscopy for monomer conversion and SEC for molar mass and dispersity (*Đ*).

Reaction condition screening: Effects of solvent, varying [TEOA], [MB^+], and tertiary amines.
For control experiments omitting DDMAT, MB^+, TEOA, or light, DMSO was added as needed to maintain a final solvent composition of 10% v/v DMSO. For varying [TEOA] and [MB^+], the corresponding stock solution volumes were adjusted accordingly. In solvent screening experiments, water was replaced with alternative solvents while maintaining 10% v/v DMSO. For polymerizations conducted without DMSO, DDMAT was added as a solid, and TEOA stock solutions were prepared in water. For DMA polymerizations using CTAs other than DDMAT, such as CETPA, CEPTA, or CMP, stock solutions were typically prepared in DMSO at concentrations ranging from 100–150 mg mL^{-1}.

Kinetic study of MB^+/TEOA photoRAFT polymerization of DMA.
The kinetic analysis followed the general procedure for MB^+/TEOA photoRAFT polymerization of DMA. The reaction mixture was irradiated under red light (640 nm, 25 mW cm^{-2}) for 4 h. At defined time intervals (0, 5, 10, 15, 30, 45, 60, 90, 120, 180, 240 min), 50 µL aliquots were taken for analysis. Monomer conversion was determined by ^1H NMR spectroscopy, and molar mass and dispersity (*Đ*) were assessed by SEC in DMF + 50 mM LiBr. Prior to SEC, samples were diluted (~1 mL) and filtered through a plug of neutral alumina to remove MB^+.

Table S1. Screening of Solvents and CTAs for the MB^+/TEOA photoRAFT Polymerization of DMA[a]

Entry	Solvent	CTA	Comment	Conv. (%)[b]	$M_{n,theory}$ (kg mol^{-1})[c]	$M_{n,app}$ (kg mol^{-1})[d]	$Đ$[d]
1	Water/DMSO (9/1 v/v)	DDMAT	Capped	90	17.8	22.4	1.14
2	Water/DMSO (9/1 v/v)	DDMAT	Open vial	87	17.2	21.8	1.14
3	PBS/DMSO (9/1 v/v)	DDMAT	Capped	95	18.9	23.6	1.13
4	DMSO	DDMAT	Capped	0	0	-	-
5	Water	DDMAT	Capped	78	15.4	19.1	1.17
6	Water	DDMAT	Open vial	84	16.7	19.1	1.17
7	EtOH/DMSO (9/1 v/v)	DDMAT	Capped	0	0	-	-
8	MeOH/DMSO (9/1 v/v)	DDMAT	Capped	0	0	-	-
9	EG/DMSO (9/1 v/v)	DDMAT	Capped	92	18.3	20.2	1.12
10	DMF/DMSO (9/1 v/v)	DDMAT	Capped	0	0	-	-
11	MeCN/DMSO (9/1 v/v)	DDMAT	Capped	0	0	-	-
12	Acetone/DMSO (9/1 v/v)	DDMAT	Capped	0	0	-	-
13	THF/DMSO (9/1 v/v)	DDMAT	Capped	0	0	-	-
14	1,4-dioxane/DMSO (9/1 v/v)	DDMAT	Capped	0	0	-	-
15	Water/DMSO (9/1 v/v)	CETPA	pH = 3	0	0	-	-
16	Water/DMSO (9/1 v/v)	CMP	pH = 3	0	0	-	-
17	PBS/DMSO (9/1 v/v)	CETPA	pH = 7	47	9.39	16.6	1.51
18	PBS/DMSO (9/1 v/v)	CMP	pH = 7	98	19.4	21.8	1.10

[a]Reaction conditions: [DMA]/[CTA]/[MB^+]/[TEOA] = 200/1/0.01/1.33, [DMA] = 3.0 M, [MB^+] = 150 μM, [TEOA] = 20 mM, in phosphate buffer (100 mM, pH = 8.0) with DMSO (10% v/v), irradiated for 6 h under red LED (640 nm, 25 mW cm^{-2}). [b]Monomer conversion determined by ^{1}H NMR spectroscopy. [c]Theoretical number-average molar masses ($M_{n,theory}$) were determined from the monomer conversion from ^{1}H NMR spectroscopy. [d]Apparent number-average molar mass ($M_{n,app}$) and dispersity ($Đ$) were determined by SEC using DMF + 50 mM LiBr relative to PMMA standards.

Table S2. Screening of Amines as Electron Donors for the MB^+/TEOA photoRAFT Polymerization[a]

Entry	Amine	Conv. (%)[b]	$M_{n,theory}$ (kg mol^{-1})[c]	$M_{n,app}$ (kg mol^{-1})[d]	$Đ$[d]
1	TEOA	95	18.8	23.7	1.16
2	MDEA	87	17.6	26.9	1.14
3	TEA	31	6.51	6.36	1.29
4	DIPEA	85	16.9	21.3	1.17
5	Me_6TREN	94	18.7	23.5	1.21
6	TPMA	41	8.14	10.8	1.34

[a]Reaction conditions: [DMA]/[DDMAT]/[MB^+]/[R_3N] = 200/1/0.01/1.33, [DMA] = 3.0 M, [MB^+] = 150 μM, [R_3N] = 20 mM,in phosphate buffer (100 mM, pH = 8.0) with DMSO (10% v/v), irradiated for 6 h under red LED (640 nm, 25 mW cm^{-2}). [b]Monomer conversion determined by ^{1}H NMR spectroscopy. [c]Theoretical number-average molar masses ($M_{n,theory}$) were determined from the monomer conversion from ^{1}H NMR spectroscopy. [d]Apparent number-average molar mass ($M_{n,app}$) and dispersity ($Đ$) were determined by SEC using DMF + 50 mM LiBr relative to PMMA standards.

Synthesis of PDMA with varying target DP (Figure 3A and Table S3).

Target degree of polymerization (DP_T) values were varied by adjusting the DDMAT/DMA ratio and MB^+ concentration to avoid excessive DDMAT/MB^+ ratios at high DP. For DP_T = 50, 100, 200, and 500, [MB^+] = 600 μM; for DP_T = 1,000 and 2,000, [MB^+] = 60 μM; and for DP_T = 10,000–20,000, [MB^+] = 26 μM. For the highest target DPs, CEPTA was used instead of DDMAT due to better performance. Polymerizations were irradiated for 10 h under red light (640 nm, 25 mW cm^{-2}), and products were analyzed by ^{1}H NMR spectroscopy and SEC.

Low molar mass PDMA samples (DP = 50 and 100) were purified via dialysis in water (regenerated cellulose membranes, MWCO = 1,000 g mol^{-1}) and then lyophilized to yield off-white, faintly blue powder. To remove MB^+ traces, dialysis against sodium citrate solution followed by water was effective. Purified $PDMA_{50}$ was used for ^{1}H NMR end-group analysis in $CDCl_3$ (Figure S5), and $PDMA_{100}$ served as a macroinitiator for NAM chain-extension to form PDMA-*b*-PNAM (Figure S4).

Table S3. Polymerization of DMA with Varying Degrees of Polymerization[a]

Entry	DP_T	$[MB^+]$ (μM)	MB (equiv)	Conv. (%)[b]	$M_{n,theory}$ (kg mol^{-1})[c]	$M_{n,app}$ (kg mol^{-1})[d]	$Đ$[d]	$M_{n,abs}$ (kg/mol)[e]
1	50	600	0.01	89	4.41	6.58	1.17	n.d.
2	100	600	0.02	97	9.65	14.7	1.14	n.d.
3	200	600	0.04	97	19.3	24.9	1.09	n.d.
4	500	600	0.10	95	47.2	73.7	1.21	n.d.
5	1,000	60	0.02	85	84.4	102	1.18	n.d.
6	2,000	60	0.02	61	120	116	1.20	n.d.
7[f]	20,000	26	0.13	68	1,350	856	1.52	1,290
8[f]	20,000	50	0.25	76	1,510	744	1.71	986
9[f]	20,000	100	0.50	88	1,750	688	1.88	860
10[g]	-	26	-	53	-	1,310	2.21	3,700

[a]Reaction conditions: $[DMA]/[CTA]/[MB^+]/[TEOA] = x/1/x/1.33$, [DMA] = 3.0 M, $[MB^+]$ = 150 μM, $[R_3N]$ = 20 mM,in phosphate buffer (100 mM, pH = 8.0) with DMSO (10% v/v), irradiated for 6 h under red LED (640 nm, 25 mW cm^{-2}). [b]Monomer conversion determined by ^{1}H NMR spectroscopy. [c]Theoretical number-average molar masses ($M_{n,theory}$) were determined from the monomer conversion from ^{1}H NMR spectroscopy. [d]Apparent number-average molar mass ($M_{n,app}$) and dispersity ($Đ$) were determined by SEC using DMF + 50 mM LiBr relative to PMMA standards. [e]Absolute number-average molar masses ($M_{n,abs}$) were determined by SEC equipped with a multi-angle light-scattering (MALS) detector using the refractive index increment (d*n*/dc) value of PDMA (d*n*/dc = 0.072 mL g^{-1} in DMF with 50 mM LiBr at 50 °C, determined assuming 100% mass recovery). [f]2-(2-Carboxyethylsulfanylthiocarbonylsulfanyl)-2-methylpropionic acid was used as the CTA when high DP_T values (10,000 – 20,000) were attempted. [g]This polymerization was performed without CTA.

Synthesis of UHMW PDMA via MB+/TEOA photoRAFT polymerization.

Stock solutions of MB^+ (0.24 mM, 0.076 mg mL-1 in H_2O), CEPTA (3.73 mM, 1 mg mL-1 in DMSO), TEOA (1.44 M, 215 mg mL-1 in DMSO) were prepared. A polymerization mixture (1.21 mL total volume) was then prepared in a 1-dram glass vial: DMA (500 μL, 4.85 mmol, 20,000 equiv), 1,3,5-trioxane (90 mg, internal standard), CEPTA stock (65 μL, 0.24 μmol, 1.0 equiv), TEOA stock (10 μL, 15 μmol, 60 equiv), MB^+ stock (133 μL, 0.03 μmol, 0.13 equiv), and H_2O (355 μL). The amount of DMSO was adjusted to achieve a final solvent composition of H_2O/DMSO (9:1, v/v). Final concentrations were: [DMA] = 4.0 M, $[MB^+]$ = 26 μM, [TEOA] = 12 mM. Prior to irradiation, a 50 μL aliquot was diluted in 550 μL D_2O to assess initial monomer content. The polymerization mixtures were irradiated under red LEDs (640 nm, 25 mW cm^{-2}), and the samples were collected for ^{1}H NMR spectroscopy and SEC analysis.

Polymerizations attempted at [DMA] = 5.0–6.0 M resulted in no conversion, likely due to insufficient water content. Similarly, no conversion was observed when DMF was used as the solvent, consistent with its structural similarity to DMA (Table S1). As discussed in the main text, effective photoinitiation with MB^+ likely requires dissociation into its monomeric form—a process that appears to occur only in water or ethylene glycol, as demonstrated in the solvent screening experiments.

Chain extension of $PDMA_{100}$-TTC with NAM.

To synthesize the PDMA-*b*-PNAM block copolymer, $PDMA_{100}$ (DP_T = 100) was first prepared using the general procedure with a feed ratio of [DMA]/[DDMAT]/[MB^+]//[TEOA] = 200/1/0.02/1.33. The crude $PDMA_{100}$ was purified by dialysis against water and freeze-dried, then used as a macroinitiator for chain extension with NAM at (DP_T = 200) under the conditions [NAM]/[$PDMA_{100}$]/[MB^+]/[TEOA] =200/1/0.02/2 in water with 10% v/v DMSO.

$PDMA_{100}$ (290 mg, 29 µmol TTC end-groups, 1.0 equiv) was dissolved in water (2.77 mL), DMF (100 µL, internal standard) and DMSO (227 µL, 10 vol%), followed by the addition of NAM (730 µL, 5.8 mmol, 200 equiv). TEOA stock solution (87 µL of 100 mg mL-1 in DMSO, 58 µmol, 2.0 equiv) and MB^+ stock solution (186 µL of 1 mg mL-1 in water, 0.58 µmol, 0.02 equiv) were then added. The polymerization mixture was irradiated under red LEDs (640 nm, 25 mW cm^{-2}) for 2 h. Final concentrations were: [NAM] = 1.5 M; [TEOA] = 39 mM; [B+] = 390 µM. The resulting block copolymer was analyzed by ^1H NMR spectroscopy and SEC.

Estimation of the concentration of terminated chains.

The concentration of terminated polymer chains ([T]) during MB^+/TEOA-mediated photoRAFT polymerization of DMA in water was estimated using an established kinetic model for reversible-deactivation radical polymerization (RDRP),[5] which relates [T] to the propagation and termination rate constants (k_p and k_t, respectively), monomer conversion (p), and reaction time (t):

$$[\mathrm{T}] = \frac{2k_{\mathrm{t}}[\ln{(1-p)}]^2}{{k_{\mathrm{p}}}^2 t}$$

Assuming the following experimental parameters:

- $[DMA]_0$ = 4.0 mol/L
- Monomer conversion p = 65% = 0.65
- Reaction time t = 240 min = 14,400 s
- k_p (DMA in water, 25 °C)[6] = 4.35×10^4 L mol^{-1} s^{-1}
- k_t (DMA in toluene, 50°C)[7] = 3.8×10^7 L mol^{-1} s^{-1}

This yields a total concentration of terminated chains:
[T] ≈ 3.65×10^{-8} mol/L = 36.5 nM

This low concentration of terminated chains ([T]) indicates negligible contribution from chain termination events during polymerization. Assuming a steady-state approximation, the number of terminated chains should equal the number of newly generated chains initiated by amine-derived radicals. This low level of termination helps explain the fairly good agreement between theoretical and absolute molecular weights, even for high molar mass samples. These findings support the high end-group fidelity observed in the chain-extension experiment.

Temporal Control of MB^+/TEOA photoRAFT polymerization of DMA.

An on/off light experiment was performed following the general procedure. Red LEDs (640 nm, 25 mW cm^{-2}) were periodically turned on for 15 min, followed by 15 min in the dark, for a total of 5 cycles (150 min). Aliquots (50 µL) were collected at the end of each light period, diluted with 550 µL D_2O, and monomer conversion was determined by ^1H NMR spectroscopy.

Polymerization under different light wavelengths.

Polymerizations were performed under various LED wavelengths, including UV (370 nm), blue (456 nm), green (527 nm), red (640 nm), and near-infrared (740 nm), using the general procedure with [DMA]/[DDMAT]/[MB^+]/[TEOA] = 200/1/0.1/1.33 and [DMA] = 3.0 M. Each reaction (1.62 mL total volume) was irradiated for 10 h at the respective wavelength and intensity (Table 2). Samples were taken at the end of the reaction for ^{1}H NMR spectroscopy to determine monomer conversion and for SEC analysis to evaluate molar mass and *Đ*. As expected, a photoiniferter-type mechanism was observed in the control experiment without MB^+ under blue light.

Sunlight-driven MB+/TEOA photoRAFT polymerization of DMA.

To assess compatibility with natural light, polymerizations were conducted under ambient sunlight using [DMA]/[DDMAT]/[MB^+]/[TEOA] = 200/1/0.003/3.33 and [DMA] = 3.0 M in PBS/DMSO (9:1 v/v, 100 mM, pH 8.0). Two reaction scales were tested: 3.3 mL and 13 mL total volumes, corresponding to 1.0 and 4.0 mL DMA, respectively. Reactions were performed outdoors at the Mellon Institute (Carnegie Mellon University) on April 23, 2025, at noon, under open-air, unstirred conditions for 1 h (Figure 4). The measured outdoor temperature near the vials reached 50 °C. Light intensities corresponding to the peak wavelengths of the Kessil LEDs used in the lab were recorded for reference to gauge the intensity of sunlight during the experiment (Table S4).

Samples were analyzed by ^{1}H NMR spectroscopy and SEC. Chain extension from crude PDMA was conducted in situ with NAM, without prior purification. Successful formation of the PDMA-*b*-PNAM block copolymer confirmed TTC end-group fidelity. Additionally, kinetic analysis was performed on the 13 mL scale by taking aliquots at 0, 15, 30, 60, 90, and 120 min (Figure 4C-E).

Table S4. Measured Sunlight Intensities at Selected Wavelengths During the Outdoor MB^+/TEOA PhotoRAFT Polymerization of DMA

Wavelength	Intensity (mW cm^{-2})
370	110
456	76
527	68
640	59

Monomer scope of the MB^+/TEOA photoRAFT polymerization.

Unless otherwise specified, polymerizations were carried out according to the general procedure using DDMAT as the CTA in H_2O/DMSO (9:1 v/v) with [DMA] = 3.0 M, [MB^+] = 150 μM, and [TEOA] = 20 mM.

Polymerizations of the following monomers followed this procedure: DMA, NAM, HEAm, NIPAM, Am, HEA, and TEGA (Table S5, entries 1–5 and 8–9).

For the methacrylamides NMMA and HPMA, four different CTAs (DDMAT, CDP, CETCPA, and CPADB) were evaluated in both water and ethylene glycol/DMSO (9:1 v/v) using [MB^+] = 300 μM and [TEOA] = 50 mM. For NMMA, CETCPA in ethylene glycol gave the best agreement between theoretical and measured molar masses and dispersities (Table S5, entry 6). HPMA was conducted at [M] = 2.0 M. Results for both methacrylamides are summarized in Table S6.

For the zwitterionic acrylate SBA, CETPA was used as the CTA in H2O/DMSO (9:1 v/v) at [SBA] = 2.0 M (Table S5, entry 10).

Methacrylate monomers HEMA and $OEGMA_{500}$ were polymerized using CPADB as the CTA. HEMA was polymerized at [M] = 3.0 M and OEGMA500 at [M] = 0.3 M, both in H_2O/DMSO (9:1 v/v) (Table S5, entries 11 and 12).

Charged or zwitterionic methacrylate monomers DMEQAMA, SPMA, CBMA, and MPC were polymerized on a 200–300 mg scale at [M] = 2.0 M in H_2O/DMSO (9:1 v/v) (Table S5, entries 13–16).

Table S5. Monomer Scope of the MB^+/TEOA-mediated PhotoRAFT Polymerization[a]

Entry	Monomer (M)	CTA	Conv. (%)[b]	$M_{n,theory}$ (kg mol^{-1})[c]	$M_{n,app}$ (kg mol^{-1})[d]	$M_{n,abs}$ (kg/mol)[e]	$Đ$[d,e]
1	DMA	DDMAT	93	18.3	22.7	-	1.15
2	NAM	DDMAT	95	26.7	23.9	-	1.14
3	HEAm	DDMAT	88	20.2	22.8	-	1.23
4	NIPAM	DDMAT	88	19.9	26.1	-	1.12
5	Am	DDMAT	70	9.93	-	21.8	1.06
6	NMMA	CETCPA	40	7.87	8.36	-	1.15
7	HPMA	CDP	59	17.0	17.8	-	1.23
8	HEA	DDMAT	72	16.6	22.3	-	1.39
9	TEGA	DDMAT	92	42.7	23.6	-	1.36
10	SBA	CETPA	90	47.8	-	36.3	1.21
11	HEMA	CPADB	70	18.3	15.3	-	1.15
12	$OEGMA_{500}$	CPADB	44	43.9	24.0	-	1.56
13	DMEAEMA	CETCPA	>99	52.6	-	62.0	1.33
14	SPMA	CETCPA	95	47.0	-	65.0	1.40
15	CBMA	CETCPA	91	41.5	-	64.1	1.13
16	MPC	CETCPA	95	56.2	-	71.7	1.08

[a]Reaction conditions: [M]/[CTA]/[MB^+]/[TEOA] = 200/1/0.01/x; [M] = 3.0 M; [MB^+] = 150 μM; [TEOA] = 20–50 mM; in phosphate buffer (100 mM, pH = 8.0) or water with DMSO (10% v/v), irradiated for 10 h under red LED (640 nm, 25 mW cm^{-2}). [b]Monomer conversion determined by ^{1}H NMR spectroscopy. [c]Theoretical number-average molar masses ($M_{n,theory}$) calculated from the monomer conversion from ^{1}H NMR spectroscopy. [d]Apparent number-average molar mass ($M_{n,app}$) and dispersity ($Đ$) were determined by SEC in DMF + 50 mM LiBr relative to PMMA standards. For polymers not soluble in DMF, aqueous SEC was used; [e]Absolute number-average molar masses ($M_{n,abs}$) and $Đ$ were determined by SEC-MALS using refractive index increment (dn/dc) measured offline in the corresponding aqueous buffer.

Table S6. Screening of CTAs and Solvents for MB^+/TEOA PhotoRAFT Polymerization of Methacrylamides (NMMA and HPMA)[a]

Entry	Monomer (M)	CTA	Solvent	[M] (M)	Conv. (%)[b]	$M_{n,theory}$ (kg mol^{-1})[c]	$M_{n,app}$ (kg mol^{-1})[d]	$Đ$[d]
1	NMMA	DDMAT	Water/DMSO (9/1 v/v)	3	10	1.99	-	-
2	NMMA	CETCPA	Water/DMSO (9/1 v/v)	3	13	2.54	-	-
3	NMMA	CDP	Water/DMSO (9/1 v/v)	3	17	3.35	-	-
4	NMMA	CPADB	Water/DMSO (9/1 v/v)	3	<5	-	-	-
5	NMMA	DDMAT	EG/DMSO (9/1 v/v)	3	33	6.46	28.4	2.41
**6**	**NMMA**	**CETCPA**	**EG/DMSO (9/1 v/v)**	**3**	**40**	**7.87**	**8.36**	**1.15**
7	NMMA	CDP	EG/DMSO (9/1 v/v)	3	25	4.85	13.3	1.20
8	NMMA	CPADB	EG/DMSO (9/1 v/v)	3	7.8	1.55	3.33	1.12
9	HPMA	CPADB	Water/DMSO (9/1 v/v)	2	69	19.8	31.8	1.20
10	HPMA	CDP	Water/DMSO (9/1 v/v)	2	72	20.6	47.8	1.23
11	HPMA	CETCPA	EG/DMSO (9/1 v/v)	2	47	13.4	32.2	1.18
**12**	**HPMA**	**CDP**	**EG/DMSO (9/1 v/v)**	**2**	**59**	**17.0**	**17.8**	**1.23**
13	HPMA	CETCPA	EG/DMSO (7/3 v/v)	2	51	14.6	18.8	1.17
14	HPMA	CDP	EG/DMSO (7/3 v/v)	2	45	12.9	28.4	1.17
15	HPMA	CETCPA	Water/DMSO (7/3 v/v)	2	56	16.1	21.6	1.12
16	HPMA	CDP	Water/DMSO (7/3 v/v)	2	60	17.1	32.8	1.16

[a]Reaction conditions: For NMMA polymerizations: [NMMA]/[CTA]/[MB^+]/[TEOA] = 200/1/0.02/3.33; [NMMA] = 3.0 M; [MB^+] = 300 μM; [TEOA] = 50 mM. For HPMA polymerizations: [HPMA]/[CTA]/[MB^+]/[TEOA] = 200/1/0.03/5.00; [HPMA] = 2.0 M; [MB^+] = 300 μM; [TEOA] = 50 mM. Both were irradiated for 10 h under red LED (640 nm, 25 mW cm^{-2}). [b]Monomer conversion determined by ^{1}H NMR spectroscopy. [c]Theoretical number-average molar masses ($M_{n,theory}$) calculated from the monomer conversion from ^{1}H NMR spectroscopy. [d]Apparent number-average molar mass ($M_{n,app}$) and dispersity ($Đ$) were determined by SEC in DMF + 50 mM LiBr relative to PMMA standards.

Monomer scope targeting DP = 10,000-20,000.

Polymerizations targeting very high degrees of polymerization (DP_T = 10,000–20,000) were carried out using CEPTA as the chain transfer agent (CTA) for acrylamide- and acrylate-type monomers, and CDP as the CTA for methacrylate-type monomers. All other reaction conditions are summarized in Table S7.

Table S7. Polymerization of Various Monomers Targeting *DP* = 10,000-20,000[a]

Entry	Monomer (M)	[M] (M)	CTA	[MB^+] (μM)	DP_T	Conv. (%)[b]	$M_{n,theory}$ (kg mol^{-1})[c]	$M_{n,app}$ (kg mol^{-1})[d]	$M_{n,abs}$ (kg/mol)[e]	$Đ^{d,e}$
1	DMA	4	CEPTA	26	20,000	68	1,350	856	1,290	1.52
2	NAM	3	CEPTA	23	10,000	97	1,130	503	1,070	1.53
3	TEGA	2	CEPTA	20	10,000	-	-	706	n.d.	1.54
4	HEAm	3	CEPTA	30	10,000	60	695	654	1,310	1.63
5	Am	5	CEPTA	25	20,000	92	1,310	-	587	1.35
6	SPMA	3	CDP	30	10,000	78	1,920	-	1,070	1.12
7	DMEQAA	3	CEPTA	30	10,000	90	2,270	-	846	1.23
8	DMEQAMA	3	CDP	30	10,000	82	2,180	-	762	1.32
9	CBMA	3	CDP	30	10,000	53	1,220	-	2,740	1.24
10	SBMA	2	CDP	20	10,000	39	1,080	-	n.d.	n.d.

[a]Reaction conditions: [M]/[CTA]/[MB^+]/[TEOA] = 10,000-20,000/1/0.1/60; [M] = 3.0 M; [MB^+] = 20-30 μM; [TEOA] = 18 mM; in phosphate buffer (100 mM, pH = 8.0) or water with DMSO (10% v/v), irradiated for 10 h under red LED (640 nm, 25 mW cm^{-2}). [b]Monomer conversion determined by ^1H NMR spectroscopy. [c]Theoretical number-average molar masses ($M_{n,theory}$) calculated from the monomer conversion from ^1H NMR spectroscopy. [d]Apparent number-average molar mass ($M_{n,app}$) and dispersity ($Đ$) were determined by SEC in DMF + 50 mM LiBr relative to PMMA standards. For polymers not soluble in DMF, aqueous SEC was used; [e]Absolute number-average molar masses ($M_{n,abs}$) and $Đ$ were determined by SEC-MALS using refractive index increment (d*n*/dc) measured offline in the corresponding aqueous buffer.

Supplementary Figures

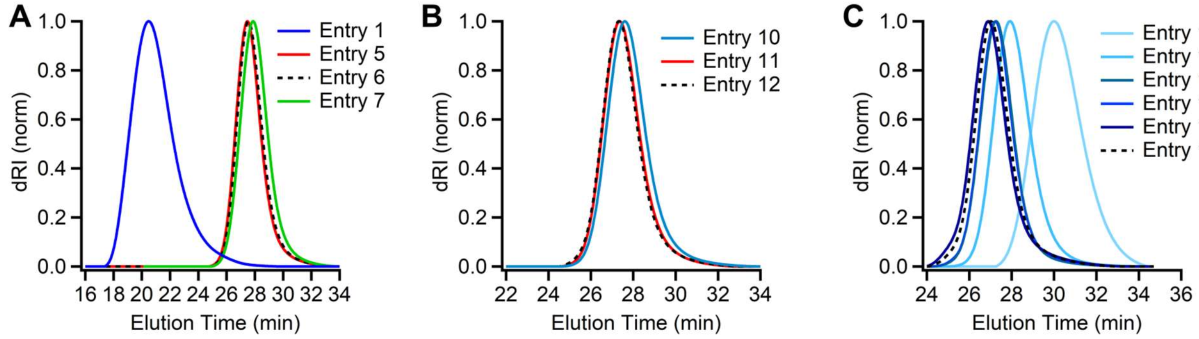

Figure S1. SEC traces corresponding to Table 1: (A) Screening of reaction conditions. (B) Effect of varying [TEOA]. (C) Effect of varying [MB^+].

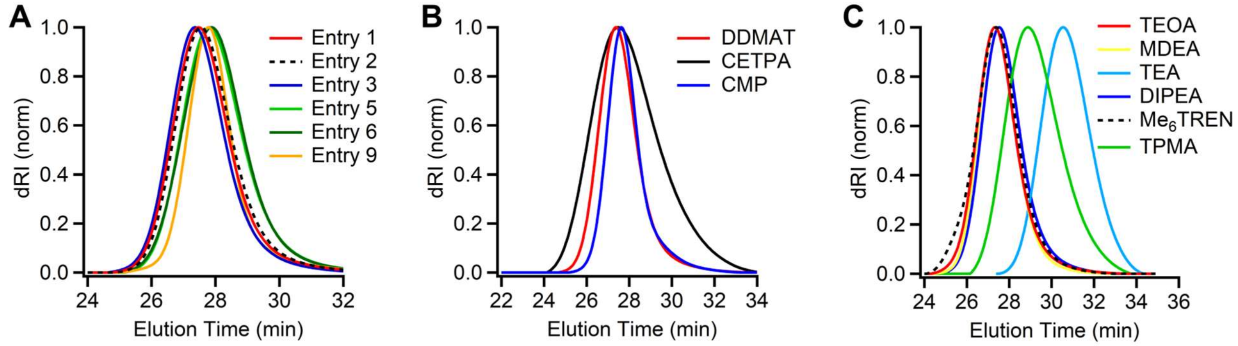

Figure S2. Overlaid SEC traces: (A) Solvent screening (Table S1). (B) Variation of CTAs (Table S1). (C) Variation of amines (Table S2).

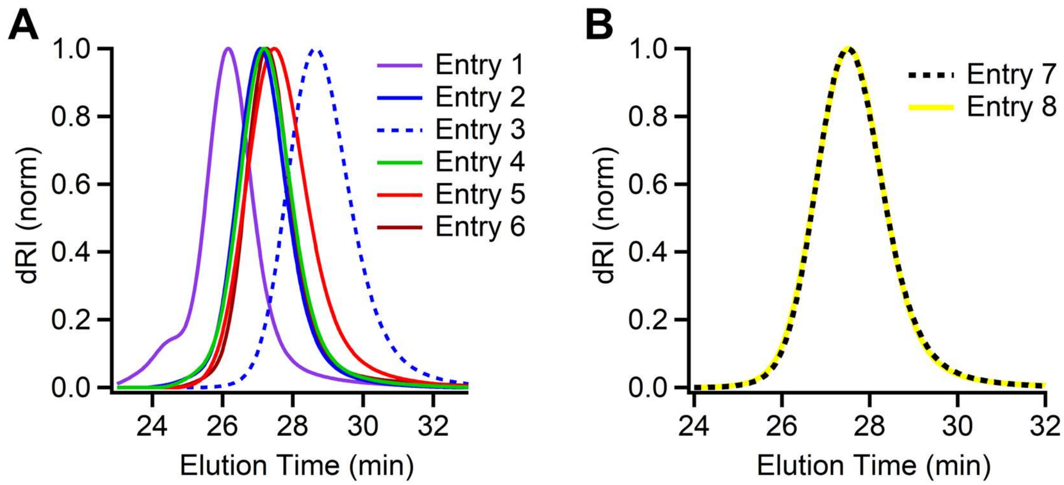

Figure S3. Overlaid SEC traces: (A) Polymerizations conducted using LEDs of different wavelengths (Table 2). (B) Polymerizations performed under ambient sunlight at two different reaction scales: 3.3 mL (entry 7, Table 2) and 13 mL (entry 8, Table 2).

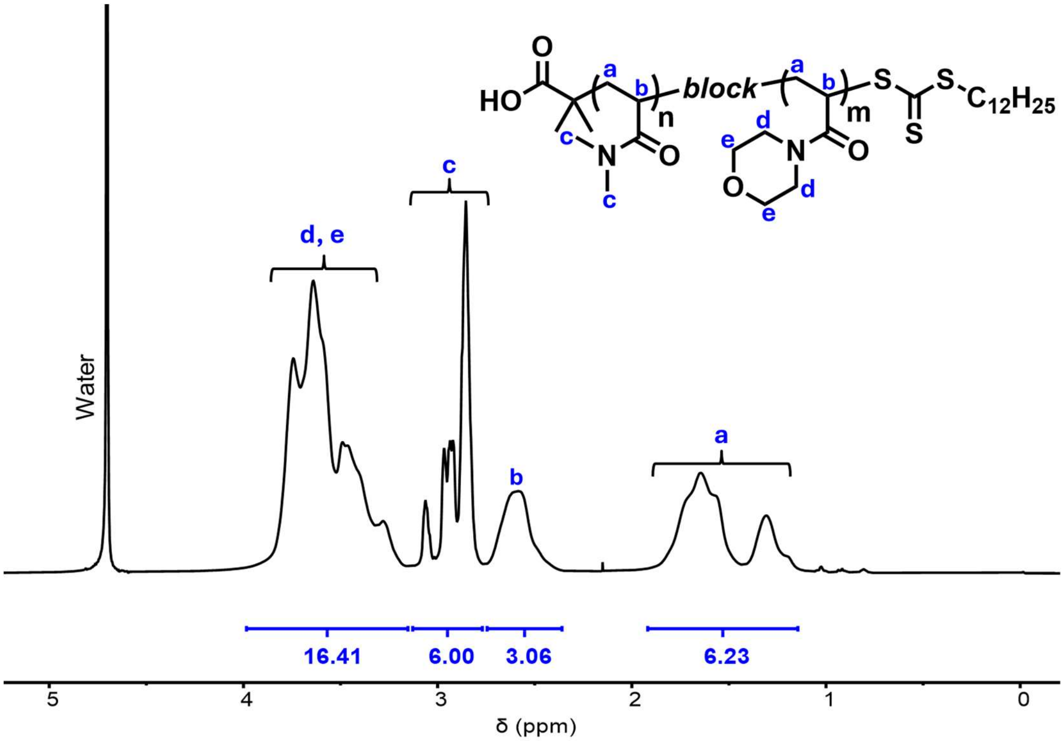

Figure S4. ^{1}H NMR spectrum (500 MHz) of PDMA-*block*-PNAM recorded in D_2O. Polymer was purified via dialysis in water (10 kDa MWCO RC membranes).

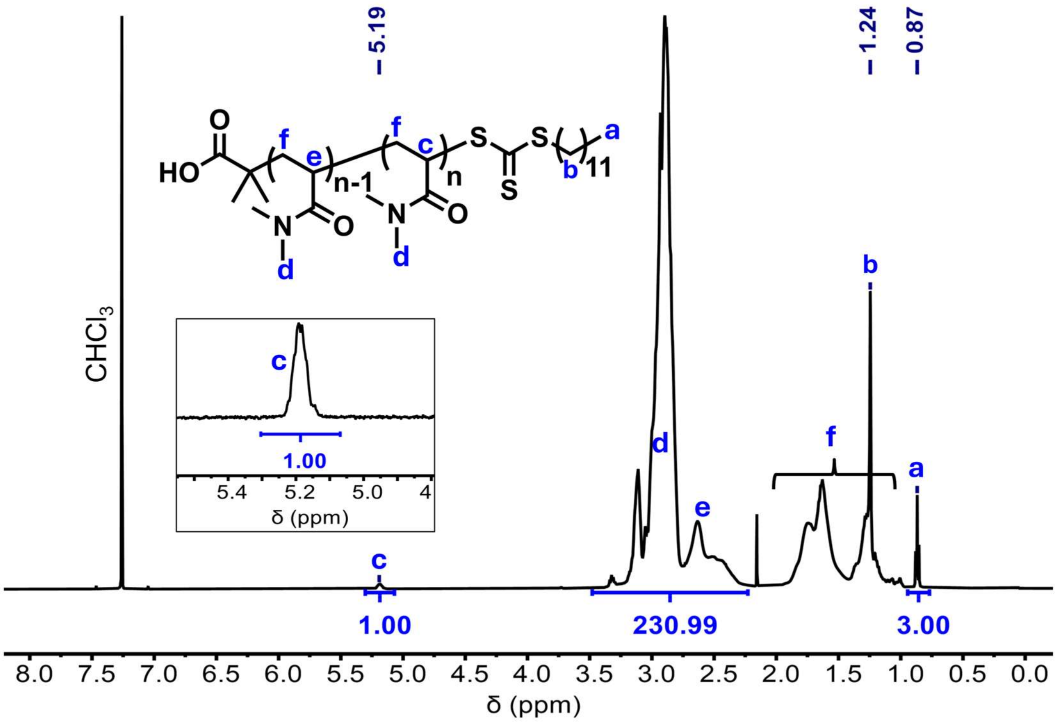

Figure S5. ^{1}H NMR spectrum (500 MHz) of low molar mass $PDMA_{50}$-TTC synthesized by MB^+/TEOA photoRAFT polymerization, recorded in $CDCl_3$.

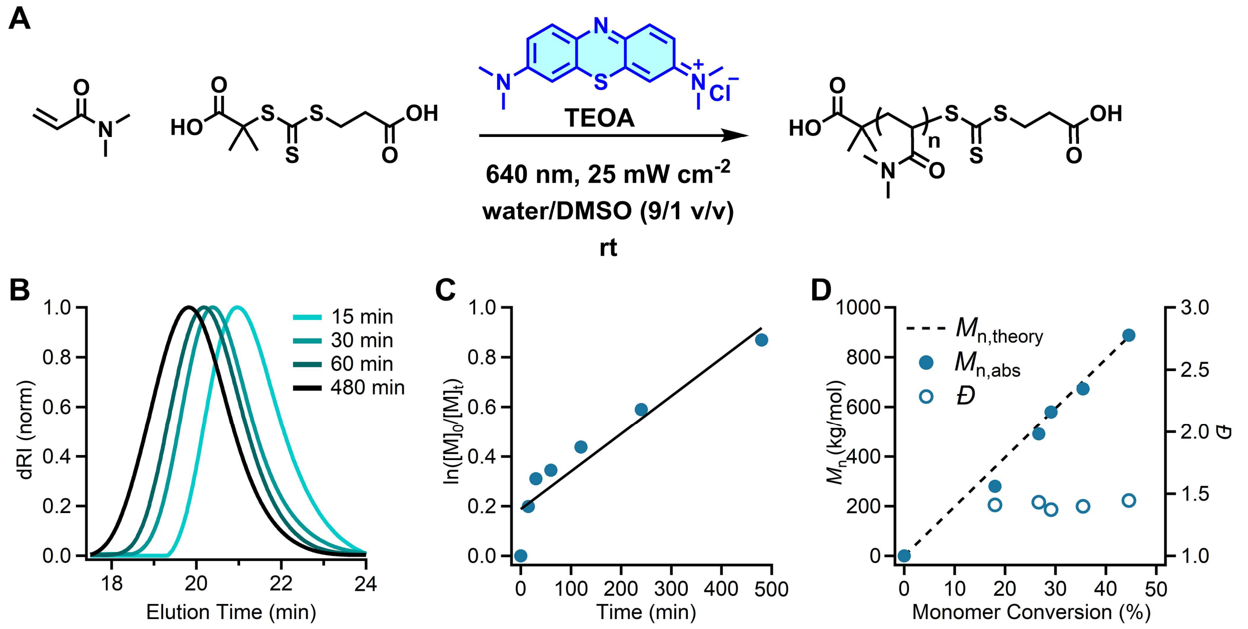

Figure S6. (A) Reaction scheme for the MB mediated RAFT polymerization of DMA targeting *DP* = 20,000. Reaction conditions: [DMA]/[CEPTA]/[TEOA]/[MB^+] = 20,000/1/60/0.15; [DMA] = 4.0 M; [TEOA] = 12 mM; [MB^+] = 30 μM. (B) Evolution of SEC traces during the MB-mediated RAFT polymerization of DMA, demonstrating monomodal shifts toward lower elution times, indicative of increasing molecular weight. (C) Pseudo-first-order kinetic plot of the polymerization. A rapid rate is observed in the first 60 min, followed by a pronounced slowdown, which we attribute to limited oxygen diffusion into the increasingly viscous medium. At high target DP and high initial monomer concentration ([DMA] = 4.0 M), the reaction mixture becomes highly viscous within 30 min and eventually forms a physical gel by extensive chain entanglements of ultra-high molar mass polymer chains. This viscosity increase further slows oxygen diffusion, reducing the rate at which the catalytic cycle can be sustained. This interpretation is supported by a deoxygenated control experiment under identical conditions, which proceeded more slowly (see Figure 2C). The black line represents a linear fit (R^2 = 0.885). (D) Plot of $M_{n,abs}$ versus monomer conversion, showing a linear increase in molecular weight with moderate dispersity ($Đ$ ≈ 1.5) up to 50% monomer conversion).

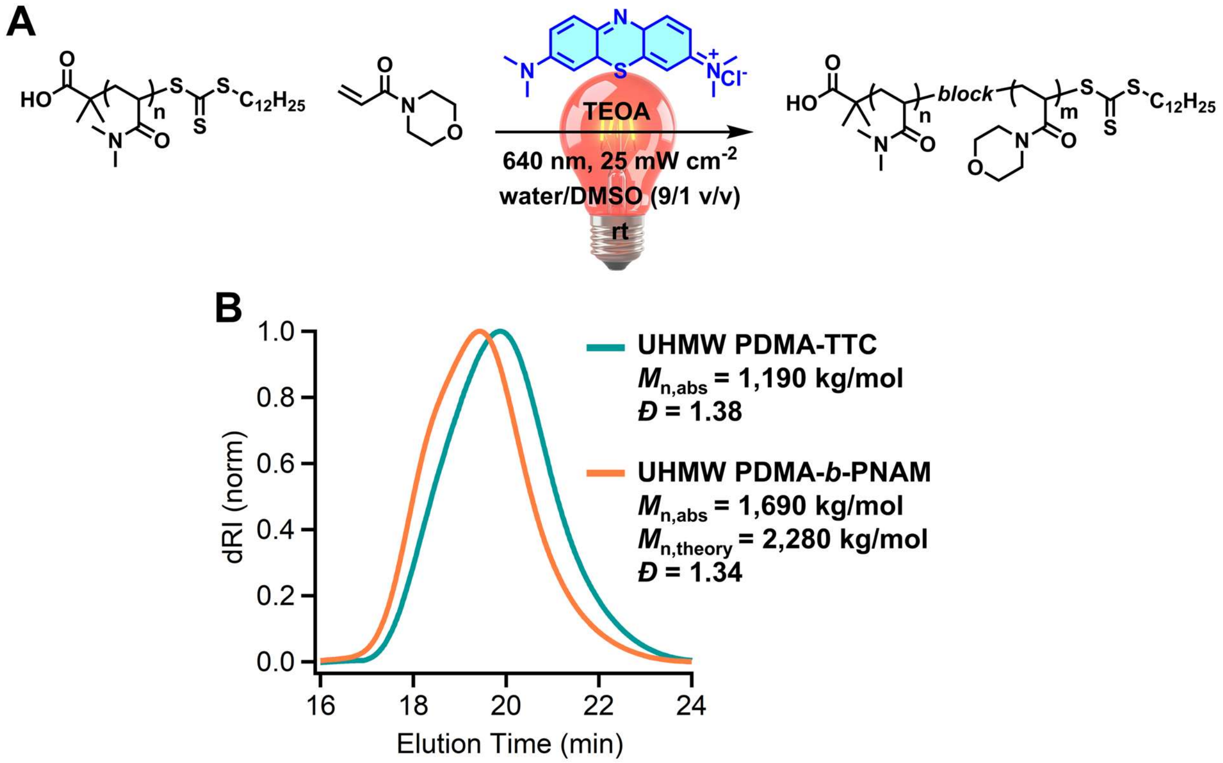

Figure S7. (A) Reaction scheme for the chain-extension of UHMW PDMA-TTC macroinitiator with *N*-acryloylmorpholine (NAM) via MB^+-mediated photoRAFT polymerization. Reaction conditions: [NAM]/[UHMW PDMA-TTC]/[TEOA]/[MB^+] = 10,000/1/60/0.1; [NAM] = 1.5 M; [TEOA] = 9.0 mM; [MB^+] = 15 µM. (B) Overlaid SEC traces of the UHMW PDMA-TTC macroinitiator and the resulting UHMW PDMA-*b*-PNAM block copolymer after 13 h of irradiation with red-light. chain-extension The chain extension achieved 77% monomer conversion, corresponding to a $M_{n,theory}$ of 2,280 kg mol^{-1}.

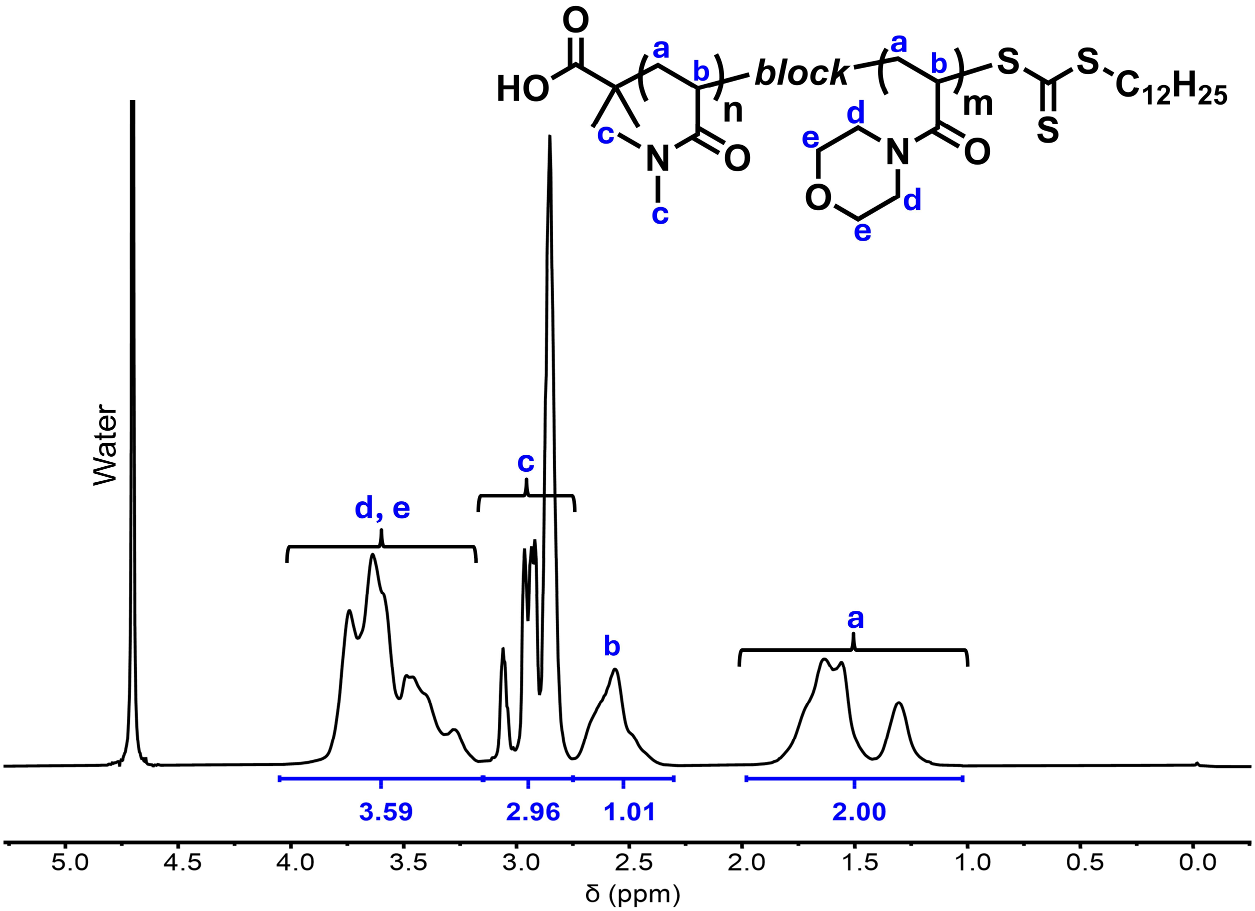

Figure S8. ^{1}H NMR spectrum (500 MHz) of UHMW PDMA-*block*-PNAM recorded in D_2O. Polymer was purified via dialysis in water (50 kDa MWCO RC membranes).

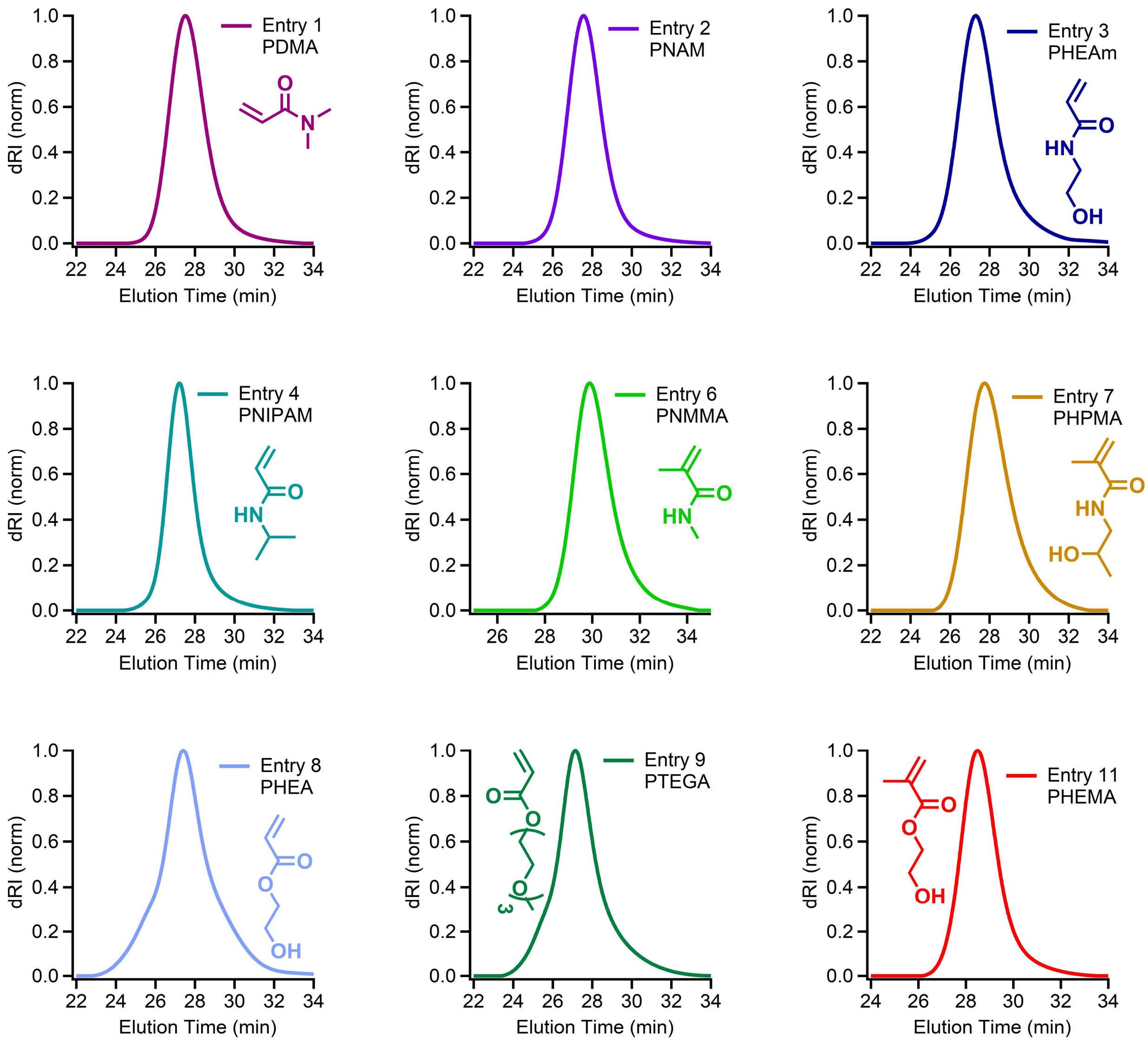

Figure S9. SEC traces of polymers ran in DMF + 50 mM LiBr (Table S5).

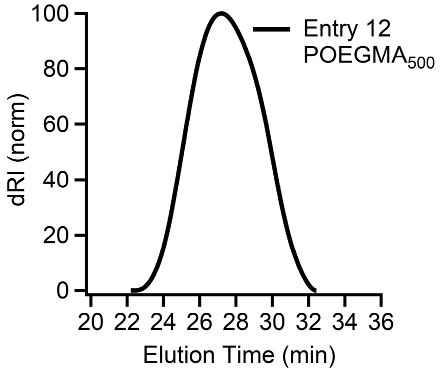

Figure S10. SEC trace of $POEGMA_{500}$ (Table S5, entry 12).

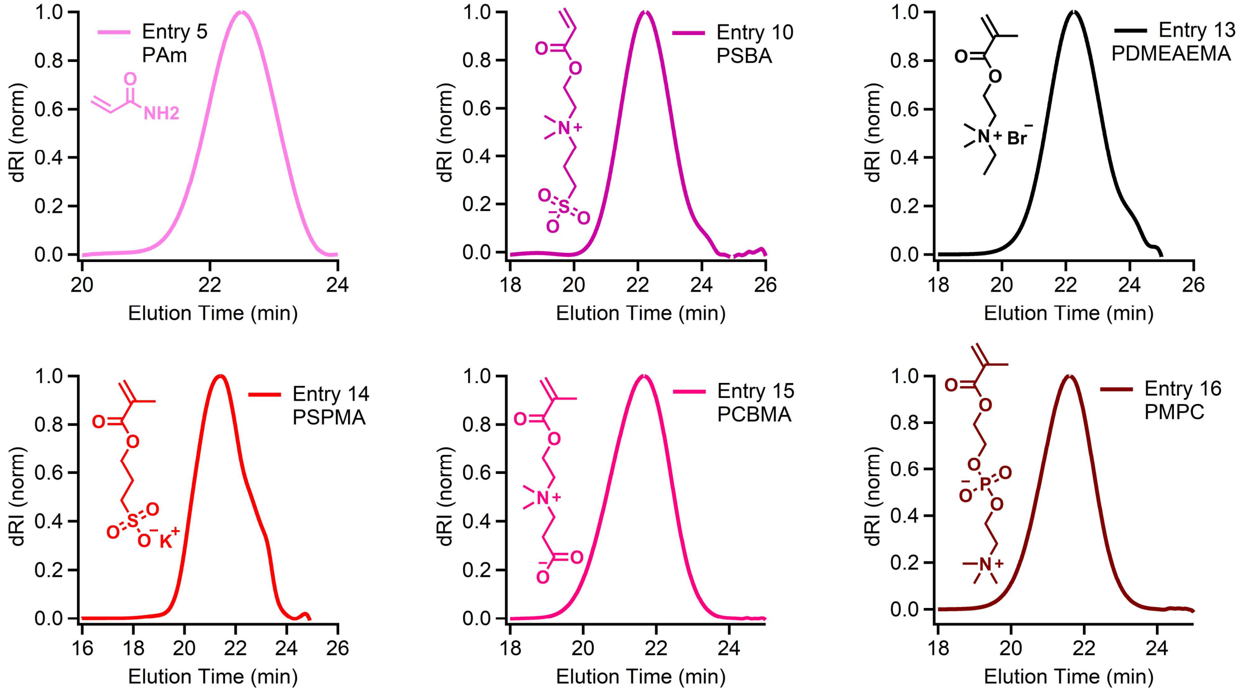

Figure S11. Aqueous SEC traces of water-soluble polymers (Table S5).

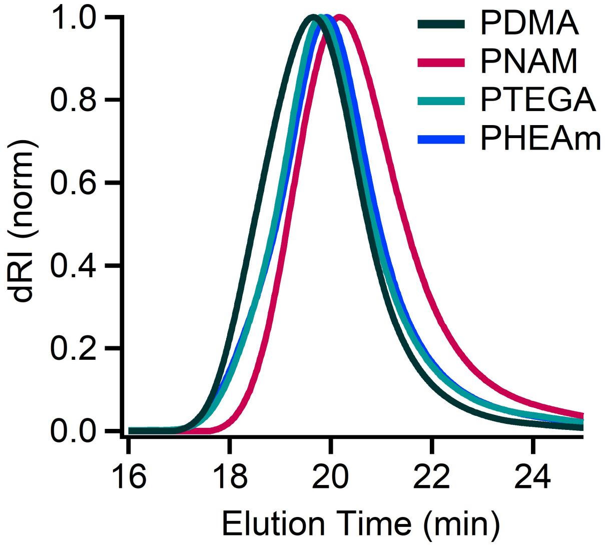

Figure S12. Overlaid SEC traces of UHMW polymers measured in DMF + 50 mM LiBr (Table S7, entries 1–4).

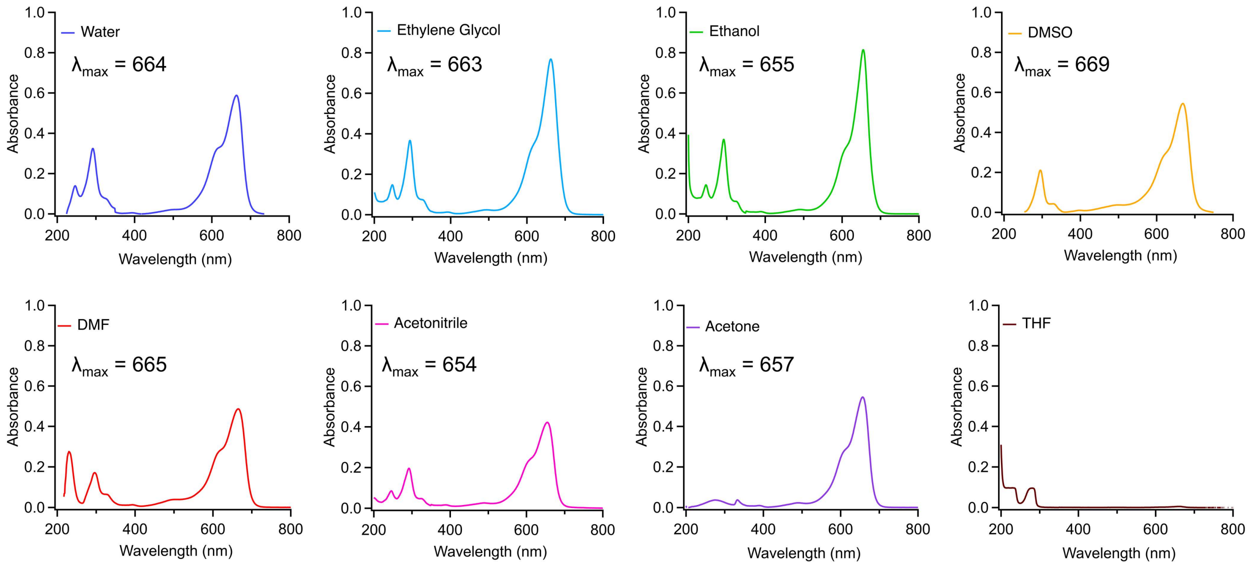

Figure S13. UV-Vis spectra of MB^+ in different solvents. Stock solutions were prepared at 1.0 mg mL^{-1} and diluted to [MB^+] = 10 μM for absorbance measurements. For THF and 1,4-dioxane, we attempted to prepare stock solutions at 0.1 mg mL^{-1}; however, MB^+ was not fully soluble, and upon dilution, no significant characteristic absorbance was detected. In the polymerization experiments conducted in THF and 1,4-dioxane, the presence of DMA, DMSO, and TEOA as co-solvents facilitated complete solubility of MB^+ at [MB^+] = 150 μM. Slight shifts in λ_{max} and differences in molar absorptivity (extinction coefficient) were observed between solvents; however, neither parameter correlated with polymerization success.

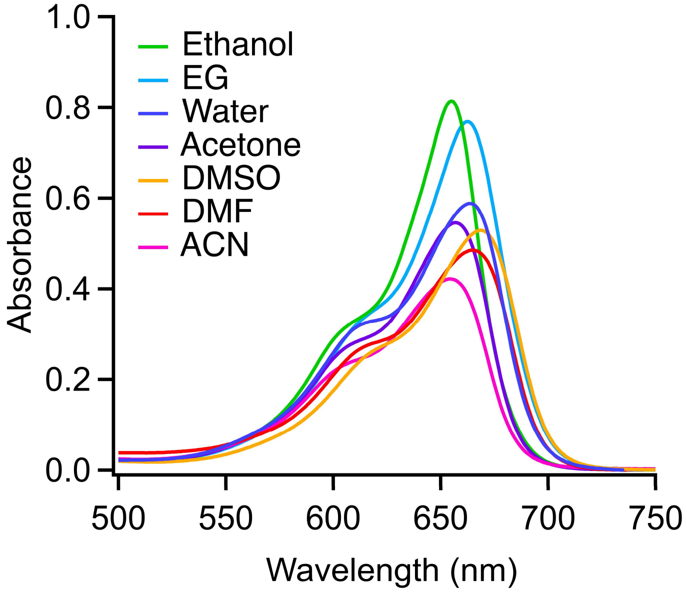

Figure S14. Overlaid UV–Vis spectra of MB^+ in different solvents ([MB^+] = 10 μM), highlighting solvent-dependent shifts in λ_{max} and variations in absorbance intensity.

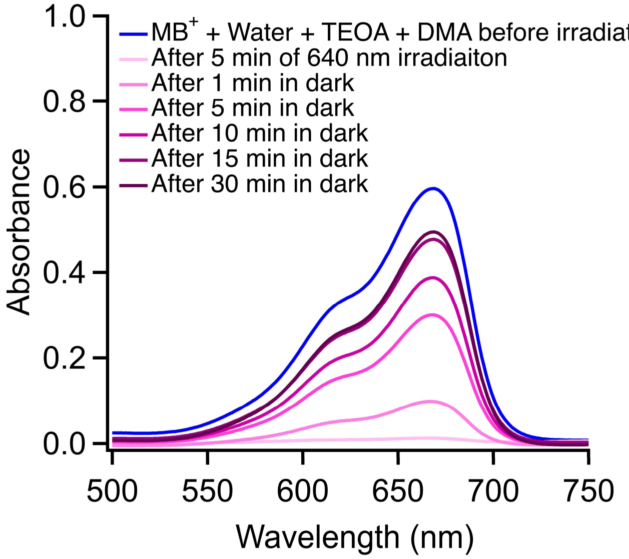

Figure S15. UV–Vis spectra of MB⁺ (40 μM) in water with TEOA and DMA before irradiation, after 5 min of 640 nm irradiation, and during recovery in the dark for up to 30 min. The decrease in absorbance at 664 nm after irradiation corresponds to reduction of MB⁺ to leucomethylene blue (LMB), while the gradual recovery in the dark indicates reoxidation of LMB to MB⁺, consistent with a reversible redox process and minimal irreversible photodegradation under these conditions.

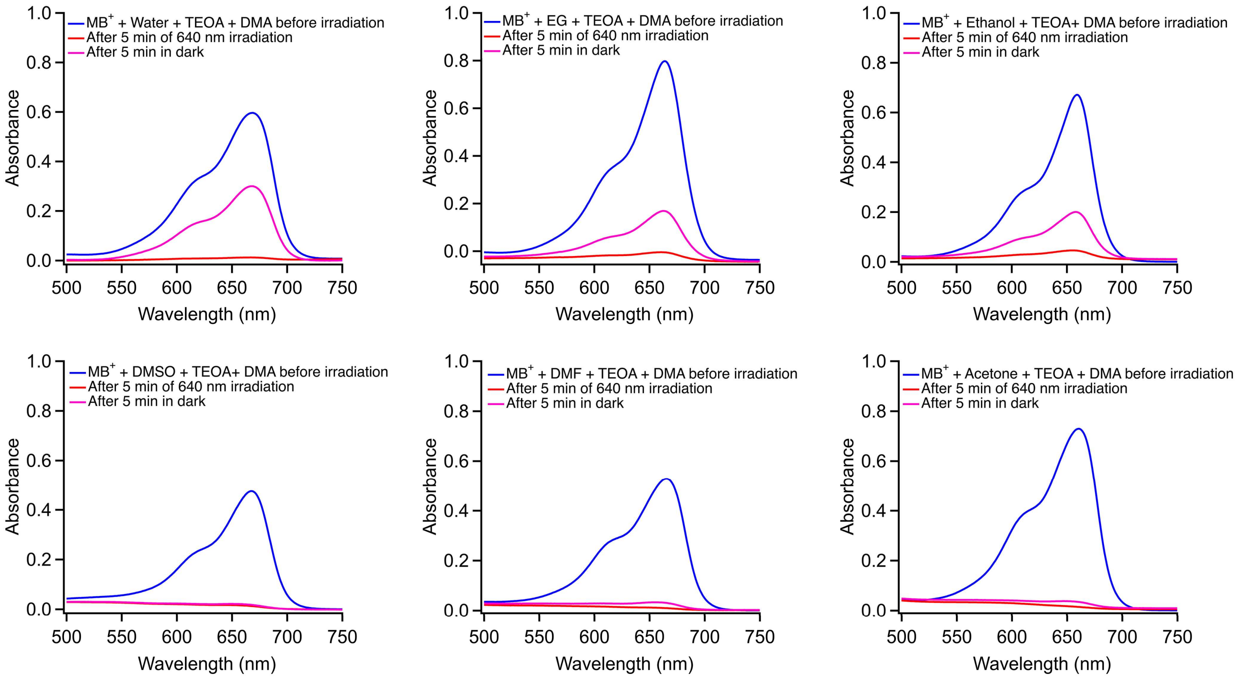

Figure S16. UV–Vis spectra of MB⁺ (10 μM) with TEOA (30 mM) and DMA (3.0 M) in various solvents before irradiation (blue), after 5 min of 640 nm irradiation (red), and after an additional 5 min in the dark (magenta). In water and ethylene glycol (EG), partial recovery of MB+ absorbance was observed after dark storage, whereas no recovery occurred in DMSO, DMF, or acetone. While there is some correlation between the absence of recovery and the lack of polymerization activity, ethanol represents an exception—exhibiting partial recovery but no polymerization under our conditions.

References

1. Lai, J. T.; Filla, D.; Shea, R., Functional Polymers from Novel Carboxyl-Terminated Trithiocarbonates as Highly Efficient RAFT Agents. *Macromolecules* **2002,** *35* (18), 6754-6756.
2. Moad, G.; Chong, Y. K.; Postma, A.; Rizzardo, E.; Thang, S. H., Advances in RAFT polymerization: the synthesis of polymers with defined end-groups. *Polymer* **2005,** *46* (19), 8458-8468.
3. Wang, R.; McCormick, C. L.; Lowe, A. B., Synthesis and Evaluation of New Dicarboxylic Acid Functional Trithiocarbonates: RAFT Synthesis of Telechelic Poly(n-butyl acrylate)s. *Macromolecules* **2005,** *38* (23), 9518-9525.
4. Murata, H.; Cummings, C. S.; Koepsel, R. R.; Russell, A. J., Rational Tailoring of Substrate and Inhibitor Affinity via ATRP Polymer-Based Protein Engineering. *Biomacromolecules* **2014,** *15* (7), 2817-2823.
5. Zhong, M.; Matyjaszewski, K., How Fast Can a CRP Be Conducted with Preserved Chain End Functionality? *Macromolecules* **2011,** *44* (8), 2668-2677.
6. Schrooten, J.; Lacík, I.; Stach, M.; Hesse, P.; Buback, M., Propagation Kinetics of the Radical Polymerization of Methylated Acrylamides in Aqueous Solution. *Macromolecular Chemistry and Physics* **2013,** *214* (20), 2283-2294.
7. North, A. M.; Scallan, A. M., The free radical polymerization of N,N-dimethylacrylamide. *Polymer* **1964,** *5*, 447-455.
